# *Gaidropsarus mauritanicus* (Gadiformes, Gaidropsaridae) a new three‐bearded rockling from a deep‐water coral ecosystem with a genetically verified biogeographical distribution of the genus and notes to its ecology and behavior

**DOI:** 10.1111/jfb.15859

**Published:** 2024-08-16

**Authors:** Alexander H. Knorrn, Lydia Beuck, David Barros‐García, Lourdes Fernández‐Peralta, André Freiwald

**Affiliations:** ^1^ Senckenberg am Meer Marine Research Department Wilhelmshaven Germany; ^2^ MARUM University of Bremen Bremen Germany; ^3^ Centro Interdisciplinar de Investigação Marinha e Ambiental (CIIMAR/CIMAR) Matosinhos Portugal; ^4^ Instituto Español de Oceanografía (IEO) Fuengirola Spain; ^5^ Department of Animal Biology, Faculty of Science University of Málaga Málaga Spain

**Keywords:** deep‐water coral habitat, micro‐CT, NE Atlantic, NW Africa, phylogenetics, species delimitation, Tanoûdêrt canyon, X‐ray, Mauritania

## Abstract

*Gaidropsarus mauritanicus* sp. nov. is described from one specimen collected using a grab sample from the Tanoûdêrt Canyon (ca. 20° N) off Mauritania at a depth of 595 m. The new species was further observed during eight remotely operated vehicle (ROV) dives along the Mauritanian slope southwards down to the Tiguent Coral Mound Complex (~17° N) in a bathymetric range between 613 and 416 m. It can be distinguished from congeners by a combination of characteristics, including large eyes (38.1% head length [HL]), large head (25.8% standard length [SL]), elongated pelvic fins (35.7% SL), low number of vertebrae (44), and coloration (pinkish with a dorsal darker brownish hue and bright blotches along the dorsal‐fin base). A species‐delimitation analysis performed with available cytochrome c oxidase subunit 1 (*COI*) sequences affiliated to the genus *Gaidropsarus* additionally supported the validity of the new species. Video analyses showed a deep‐water coral‐associated and protection‐seeking behavior, which may explain why the species has remained undescribed until now. Additional ROV footage from separate deep‐water coral sites in the North Atlantic and Mediterranean Sea further highlights the ecological behavior and hidden diversity of bathyal three‐bearded rocklings. Here, we additionally discuss the biogeographical distribution of all genetically verified *Gaidropsarus* spp. in combination with genetic data and morphological characters. *G. mauritanicus* sp. nov. is closely related to a species from Tasmania (43° S), a geographical point furthest among the studied samples, which may hint to an important influence of (paleo‐) oceanography on the distributions of *Gaidropsarus* species.

## 1 INTRODUCTION

The order Gadiformes encompasses some of the most important species for today's commercial fisheries. There are also smaller species, such as the three‐bearded rocklings of the genus *Gaidropsarus* Rafinesque, 1810, with nearly no commercial value but a remarkable distribution range. The phylogenetic position of three‐bearded rocklings has been subject to much scientific debate, resulting in various classifications, including placement within different family groups such as Gaidropsaridae (Howes, 1991; Roa‐Varón et al., 2021), Gadidae (Endo, 2002; Nelson et al., 2016; Roa‐Varón & Ortí, 2009; Teletchea et al., 2006), or Lotidae (Van der Laan et al., 2014). The systematics and diversity of *Gaidropsarus* remain unclear, although its placement within the family Gaidropsaridae, along with *Ciliata* and *Enchelyopus*, is well supported by Roa‐Varón et al. (2021). Some members of the genus have since been synonymized, such as *Gaidropsarus biscayensis*, which is now considered a synonym of *Gaidropsarus macrophthalmus*, or *Gaidropsarus guttatus*, which is considered a synonym of *Gaidropsarus mediterraneus* (Barros‐García et al., 2018; Orsi Relini & Relini, 2014). The genus currently comprises 13 recognized species, including *Gaidropsarus argentatus* (Reinhardt, 1837), *Gaidropsarus capensis* (Kaup, 1858), *Gaidropsarus ensis* (Reinhardt, 1837), *Gaidropsarus gallaeciae* Bañón et al., 2022, *Gaidropsarus granti* (Regan, 1903) 2022, *Gaidropsarus insularum* Sivertsen, 1945, *G. macrophthalmus* (Günther, 1867), *Gaidropsarus mauli* Biscoito & Saldanha, 2018, *G. mediterraneus* (Linnaeus, 1758), *Gaidropsarus novaezealandiae* (Hector, 1874), *Gaidropsarus pakhorukovi* Shcherbachev, 1995, *Gaidropsarus parini* Svetovidov, 1986, and *Gaidropsarus vulgaris* (Cloquet, 1824).

Rocklings display a wide range of ecological adaptations, inhabiting environments from the intertidal zone to deep‐sea habitats and from arctic to temperate and subtropical waters (Bañón et al., 2022). Members of the genus are characterized by a slender, elongated body, a chin barbel, nostril barbels, three barely separated dorsal fins, a long anal fin, and a lateral line that is interrupted along the entire length of their bodies (Cohen et al., 1990; Svetovidov, 1986a). However, Svetovidov (1986b) notes that distinguishing between different species of *Gaidropsarus* can often be challenging.

Due to a lack of comprehensive rockling collections (Balushkin, 2009) and no comprehensive barcode library for the genus, the taxonomy of this group is incomplete and inadequately understood. Recent molecular studies (Barros‐García et al., 2022) have revealed inconsistencies with the morphology‐based taxonomic concept, identified potential new species, and indicated possible synonyms for some shallow‐water species. In this study, we confirm the presence of a new species, previously referred to as *Gaidropsarus* sp. 3 in Barros‐García et al. (2022), and incorporate it into the existing knowledge regarding the genus, contributing to the advancement of our understanding of rockling taxonomy. The additional analysis of video material from a remotely operated vehicle (ROV) has further allowed the characterization of its habitat and behavior. In addition, we have conducted species delimitation analyses for all genetically validated *Gaidropsarus* spp. records available to test this new species hypothesis and compare it with morphological characters. We also mapped all genetically verified records with exact coordinates and extracted the depth for each record from GEBCO (2023). This has allowed us to characterize biogeographical and bathymetrical patterns on species level and discuss the role of deep‐water coral ecosystems in relation to deep‐water *Gaidropsarus* species.

## 2 MATERIALS AND METHODS

### 2.1 Study and sampling area

From October to November 2010, the R.V. *Maria S. Merian* (MSM) Cruise 16/3 PHAETON carried out targeted ROV surveys and sampling off Mauritania (Westphal et al., 2012). Twelve ROV dives were made along an N–S stretch of approximately 353 km (following the 500‐m‐depth contour). They were conducted on submarine canyon flanks and open‐slope mound complexes. The spatial coverage stretched from off Cape Blanc (20°14.840′ N) southwards to latitude 17°08.203′ N. The holotype was collected in the Tanoûdêrt Canyon via grab with a diverse assemblage of live deep‐water fauna on a dead coral framework (Station Geo‐B: 14802‐1; 20°14.791′ N 17°40.188′ W) at a depth of 595 m (Gil et al., 2020; Matsuyama et al., 2015; Sampaio et al., 2022; Westphal et al., 2012).

The collected specimen was promptly photographed while alive and subsequently preserved in 96% EtOH plus 1% MEK. The preserved holotype underwent further documentation and imaging using a Nikon D700 camera and a digital light microscope (Keyence VHX—1000D). A tissue sample was obtained from the right flank for subsequent DNA sequencing of the cytochrome c oxidase subunit 1 mitochondrial gene (*COI*) (Bold‐ID GSRUS149‐16), as outlined in Barros‐García et al. (2018), where the specimen was initially referred to as *Gaidropsarus* sp. 3.

### 2.2 Morphological measurements

All measurements and fin ray counts were conducted on the left side of the specimen, following the methodology outlined by Svetovidov (1986a, 1986b). The terminology for the lateral line system employed in this study was adopted from Böhlke (1989). To ensure accuracy, measurements and fin ray counts were obtained by observing the left side of the fish under a stereomicroscope at a magnification of 20× and by using X‐ray photography. Morphological features were carefully compared with the comprehensive review of the genus *Gaidropsarus* provided by Bañón et al. (2022) and Biscoito and Saldanha (2018), which included data on morphological characters, distribution, and coloration reported in ichthyological literature as well as measurements obtained by Bañón et al. (2022).

### 2.3 Micro‐CT scanning and x‐ray photography

High‐resolution X‐ray micro‐computer tomography of the holotype was performed with a Zeiss X‐Radia Context at the Senckenberg Natural History Collections in Dresden, Germany. The scanning parameters were as follows: duration 3.56 h, 2401 projections, eight frames and an exposure time of 0.6 s, voxel size 9.1 μm, source voltage and power 70 kV and 6 W, respectively, with a filter LE1. Subsequently, the volumetric data obtained from the scan were post‐processed using the Thermo Scientific Amira 3D Pro and XFiber software. Additional radiographs were obtained using a Fxitron LX‐60 unit.

### 2.4 Phylogenetic analyses

All publicly available *COI* sequences belonging to *Gaidropsarus* were mined from the repositories BOLD‐Systems and GenBank (August 2023) (Ratnasingham & Hebert, 2007; Sayers et al., 2022). After cross‐referencing and validation, the dataset comprised 204 *Gaidropsarus* sequences (see supplemental material). From these, unique haplotypes were retrieved for further analyses (*n* = 47). In addition, a sequence of Atlantic cod (BNSF005‐11 *Gadus morhua*) was used as an out‐group for phylogenetic inference. The final alignment comprised 48 sequences with a length of 651 nucleotides.

The optimal partition strategy for the data and substitution model was estimated with PartitionFinder v1.1.1 (Lanfear et al., 2012) and jModelTest2 (Darriba et al., 2012; Guindon & Gascuel, 2003), respectively. Therefore, the Hasegawa model (HKY) with a Gamma distribution (+G) and a single partition was used as the prior for phylogenetic analyses.

Bayesian inference was carried out using BEAST V2.5.2 with two runs of 10 million generations sampling each 1000 and four independent chains. The convergence of the analyses (Effective Sample Size values >200) was confirmed with TRACER.1.7.0 (Rambaut et al., 2018). The final consensus tree was obtained after discarding the first 25% of the trees and a posterior probability limit of 0.9 in TreeAnnotator v.2.4.5 and visualized with FigTree v.1.4.3 (http://tree.bio.ed.ac.uk/software/figtree/).

A maximum likelihood (ML) analysis was carried out using the IQ‐tree tool present in XSEDE (3.2.6) in the CIPRES portal with 1.000 rapid bootstrap replicates (Miller et al., 2010). The tree with the highest ML was selected as input for the Poisson Tree Processes (PTP) analyses.

### 2.5 Species delimitation analyses

Six different species DNA delimitation analyses were carried out in the *Gaidropsarus* dataset to test the validity of *Gaidropsarus mauritanicus* sp. nov. Two of them were distance based; the Barcode Index Number (BIN) is available at BOLD systems (https://www.boldsystems.org/) (Ratnasingham & Hebert, 2013), and the Assemble Species by Automatic Partitioning (ASAP) is available at https://bioinfo.mnhn.fr/abi/public/asap/ (Puillandre et al., 2021). The BIN data regarding *Gaidropsarus* sequences were obtained from BOLD systems (supplemental material). A web server was used to estimate the best data partition with ASAP. To this end, p‐distance was applied to the *COI* alignment without out‐group. The best partition was selected considering the ASAP score and the threshold distance.

Four different approaches were used for delimitation analyses based on phylogenetic trees: bPTP, a variation of PTP that adds Bayesian support values to delimited species (Zhang et al., 2013); mPTP; a multi‐rate PTP that includes different levels of intraspecific diversity (Kapli et al., 2017); GMYC identifies the time threshold that defines coalescent or speciation processes on ultrametric trees (Fujisawa & Barraclough, 2013; Pons et al., 2006); and mGMYC, which is identical to the former, but assuming several coalescence processes across the tree (Monaghan et al., 2009). The webserver (https://species.h-its.org/ptp/) was used for bPTP and (https://mptp.h-its.org/#/tree) for the mPTP, using the ML tree as input for both after removing the out‐group to optimize the results (Zhang et al., 2013). Both GMYC and mGMYC analyses were executed using the R Package “splits” with the function “gmyc” and the functions “method = single” and “method = multiple” for both analyses (Fujisawa & Barraclough, 2013).

### 2.6 Data analysis of ROV dives

All dives were conducted with the ROV Sperre AS SUB‐fighter 7500 DC, operated by Sven Lovén (Centre for Marine Infrastructure at the University of Gothenburg, Sweden). The ROV was equipped with one video camera (720 × 576 pixels), a high‐definition video camera, a still camera, halogen lights, sonar, CTD, an oxygen meter (optode), two laser points (scaling: 5 cm), a five‐function manipulator, and a sampler box. A ROV‐based positioning system was used, and the video footage was linked to the navigation track using time code. During the dive, the video signal was stored in digital Quick Time MPEG‐4 format (*.mov) with a resolution of 1280 × 720 pixels, a bit rate of 100 MB/s and in sequences of each about 5 min. Minifilms with an interval of 1 s were created for all dives. The ROV navigation track was cleaned using the ArcGIS extension Adelie of IFREMER through manual cleaning and Gaussian smoothing. Observation records of *Gaidropsarus* individuals were annotated with habitat type, depth, and behavior. Video clips showing its behavior were produced with Pinnacle Studio 21 (see supplement material S2 & S3).

### 2.7 Mapping of validated records

To map the geographic distribution of *Gaidropsarus* species, the specimens with both a validated species assignation and metadata containing exact coordinates were selected. Thus, the final sub‐dataset contained in total 186 *Gaidropsarus* sequences (see supplemental table). After a manual correction of the coordinates due to technical issues (compare row I with K and J with L in Appendix Table S1), all records were plotted in ArcMap (see also Table 3 and Figure 9).

## 3 RESULTS

Class: Osteichthyes Huxley, 1880.

Order: Gadiformes Goodrich, 1909.

Family: Gaidropsaridae Jordan & Evermann, 1898.

Genus: *Gaidropsarus* Rafinesque, 1810.

Species: *Gaidropsarus mauritanicus* sp. nov. Knorrn, Beuck, & Freiwald, 2024.

### 3.1 Species identification

The specimen can be affiliated with the genus *Gaidropsarus* Rafinesque 1810 as it displays the morphological characters previously described by Cohen et al. (1990). These characteristics include the presence of three barely separated dorsal fins, with the first possessing only one single thickened and unsegmented ray, the second having several small and unsegmented rays in a fleshy elongated groove, and the third with segmented rays in an elongated fin. Additionally, the species has three prominent barbels: one located on the chin and one at each anterior nostril on the snout, in addition to a prominent anal fin and an interrupted lateral line running along the entire length of the body.

*G. mauritanicus* sp. nov. can morphologically be differentiated from all other valid *Gaidropsarus* species by having 44 vertebrae, which distinguishes it from *G. argentatus*, *G. ensis*, *G. mediterraneus*, *G. vulgaris*, *G. mauli*, *G. insularum*, *G. novaezealandiae*, *G. pakhorukovi*, and *G. parini*. Additionally, *G. mauritanicus* sp. nov. has a longer pelvic fin (35.7% standard length [SL]) than species with fewer vertebrae (*G. gallaeciae*, *G. granti*, *G. macrophthalmus*, *G. capensis*); a shorter pectoral fin (13.8% SL) than *G. argentatus*, *G. gallaeciae, G. ensis, G. macropththalmus*, and *G. mauli*. Notably, having fewer than 45 vertebrae, *G. mauritanicus* sp. nov. forms a clade with *G. gallaeciae*, *G. macrophthalmus*, *G. capensis*, and *G. granti*. An overview of the most outstanding morphological characteristics between *G. mauritanicus* sp. nov. and every other valid species is given in Table 3.

### 3.2 Holotype

SMF: 39643 (72.61 mm total length [TL], 62.49 mm SL); Mauritania; Tanoûdêrt Canyon; 20°14.791′ N; 17°40.188′ W; 595‐m water depth associated with deep‐water coral fauna; November 3, 2010, at 10:33 p.m. UTC; André Freiwald & Lydia Beuck leg.; R.V. *Maria S. Merian* (MSM) 16/3 grab sample; sta. no. Geo‐B: 14802–1; GenBank‐ID: KY370534.

### 3.3 Diagnosis

*G. mauritanicus* sp. nov. differs from all other 13 valid *Gaidropsarus* species by the combination of the following characteristics: large eyes (38.1% HL) versus small eyes, relatively large head (25.8% SL), elongated pelvic fin (35.7% SL) versus medium‐sized pelvic fin, small pectoral fin (13.8% SL) versus enlarged pectoral fin, 44 vertebrae, coloration (pinkish with a darker brownish hue around the dorsal side and brighter dots around the dorsal‐fin bases), and habitat preference (deep‐water coral ecosystems). For detailed comparisons between the species, see Tables 2 and 3.

## 4 DESCRIPTION

### 4.1 Body morphology

Morphometric and meristic characteristics of *G. mauritanicus* sp. nov. are given in Table 1. For a general view of the specimen, see Figures 2, 3, 4. Body elongated and laterally compressed, head large and moderately compressed (25.8% SL) with dorso‐laterally positioned large eyes (38.1% head length [HL]). Mouth Inferior with slightly longer upper jaw. Lower jaw extends to posterior end of eye. Snout shorter than eye diameter. Anterior nostrils tubular, with one barbel directly behind each nostril. A third barbel positioned at the tip of the lower jaw. Total vertebrae 44: 13 precaudal and 31 caudal (including the urostyle).

**TABLE 1:** Morphometric measurements and meristic counts from the holotype of *Gaidropsarus mauritanicus* sp. nov.

	*Holotype*		
	Mm	% SL	% HL
Morphometrics			
Total length (TL)	72.61	‐	‐
Standard length (SL)	62.49	‐	‐
Head length (HL)	16.121	25.8	‐
Snout length	4.099	6.5	25.4
Upper jaw length	6.013	9.6	37.3
Lower jaw length	5.739	9.2	35.6
Eye (horizontal diameter)	6.142	9.8	38.1
Eye (vertical diameter)	4.174	6.7	25.9
Interorbital distance	3.068	4.9	19
Postorbital length	8.189	13.1	50.8
Pectoral‐fin length	8.602	13.8	‐
Pectoral‐fin height	4.616	7.4	‐
Pelvic‐fin length	22.283	35.7	‐
Prepelvic‐fin length	13.310	21.3	‐
First predorsal length	14.248	22.8	‐
Second dorsal‐fin base length	10.561	16.9	‐
Third dorsal‐fin base length	35.244	56.4	‐
Third predorsal length	24.871	39.8	‐
Anal‐fin length	30.495	48.8	‐
First ray of first dorsal‐fin length	4.017	6.4	24.9
Body height at anus	0.998	1.6	6.2
Body height at pectoral‐fin base level	0.910	1.5	‐
Right nasal barbel length	2.208	3.5	‐
Left nasal barbel length	2.646	4.2	‐
Chin barbel length	3.238	5.2	‐
Caudal peduncle length	3.344	5.4	‐
Caudal peduncle height	3.844	6.2	‐
Body depth	6.124	9.8	‐
Meristics			
First dorsal‐fin rays	1		
Second dorsal‐fin rays	46		
Third dorsal‐fin rays	57		
Anal‐fin rays	53		
Pectoral‐fin rays	24		
Pelvic‐fin rays	7		
Caudal‐fin rays	35		
Gill rakers (outer row)	7–8		
Vertebrae	13 + 31 = 44		

### 4.2 Fin morphology

D1 1, D2 46, D3 57, P 24, V 7, A 53, C 35. Fin length, proportions, and meristics are given in Table 1. Fin ray of D1 fleshy and longer than the fin rays of D2. Broad pectoral fin and a strongly elongated V. P reaches D3. There is no connecting membrane between D2 and C. A originates posterior of origin of D3. Rounded C which is longer than tall.

### 4.3 Lateral line system

**FIGURE 1:**
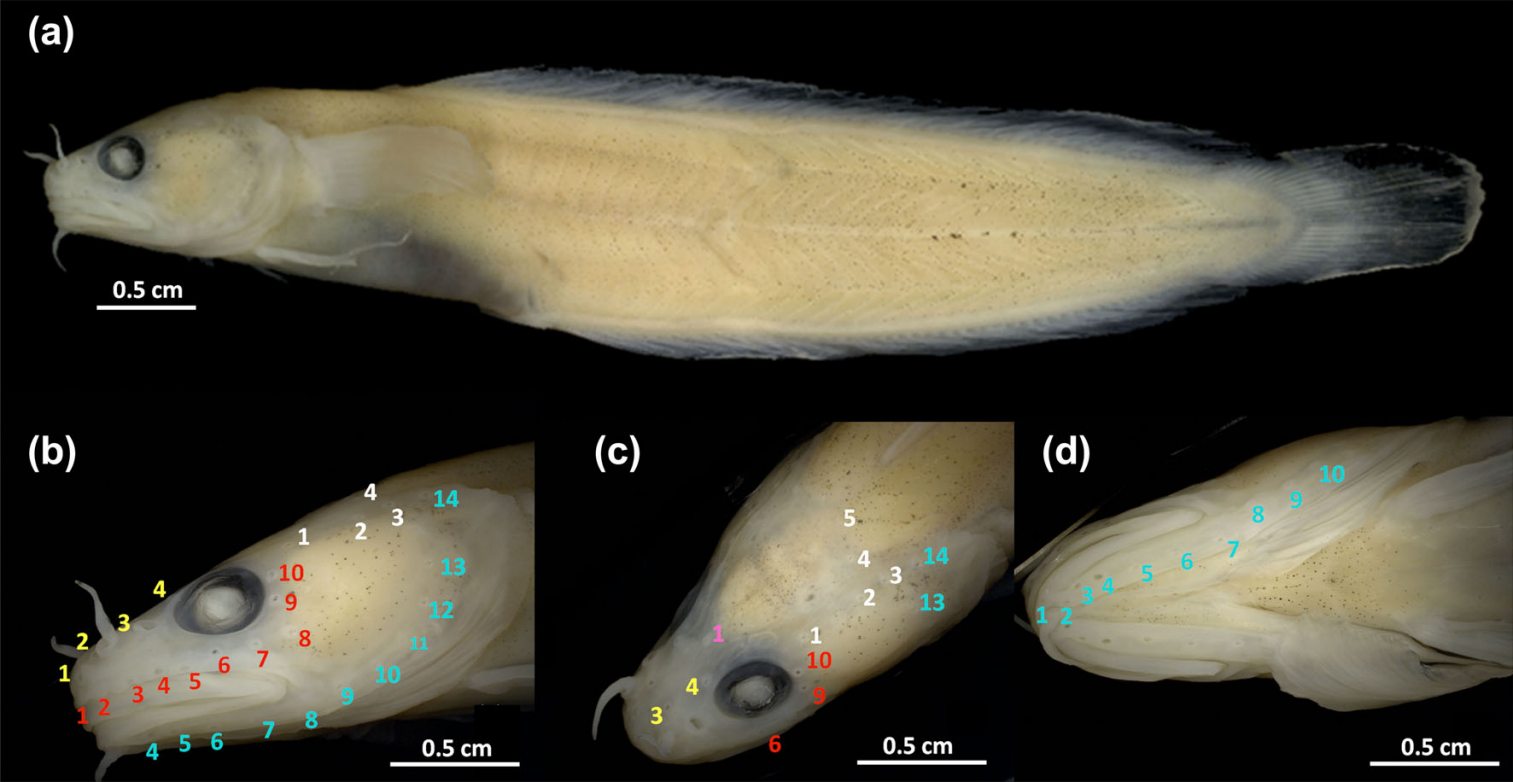
Photography of preserved holotype of *Gaidropsarus mauritanicus* sp. nov. (a) Overview, lateral view (left side). (b–d) Detailed view of head with head lateral line system of *G. mauritanicus* sp. nov.: supraorbital canal (yellow), infraorbital canal (red), supraorbital commissure (pink), temporal canal (white), and preoperculo‐mandibular canal (blue).

Lateral line is weakly pronounced and consists of five small segments with a pore at the end. The head lateral line system is strongly pronounced and can be seen in Figure 1. Supraorbital canal consists of four pores: the first one close to the tip of the snout, the second slightly behind the first and on the same level as the anterior nostril, the third one higher on the level between the anterior and posterior nostrils, and the fourth at the anterior base of the eye. One single pore is present between the eyes at the supraorbital commissure. Temporal canal possesses five pores: the first postorbital behind the eye, with the second through fifth in a row reaching from the end of the operculum to the start of the lateral line. Supratemporal canal possesses one pore on each side of the face in dorsal direction, slightly behind the fourth temporal canal pore. Infraorbital canal has 10 pores: the first 6 pores are located along the anterior margin of the upper labial fold, and numbers 7–9 are located underneath the eye. Number 10 is located in a postorbital position. Preoperculo‐mandibular canal has 14 pores: first in front of the barbel, second to six underneath the lower jaw in a single row, seventh and eighth closely together at the end of the lower jaw, and tenth to fourteenth in a preopercular position.

**FIGURE 2:**
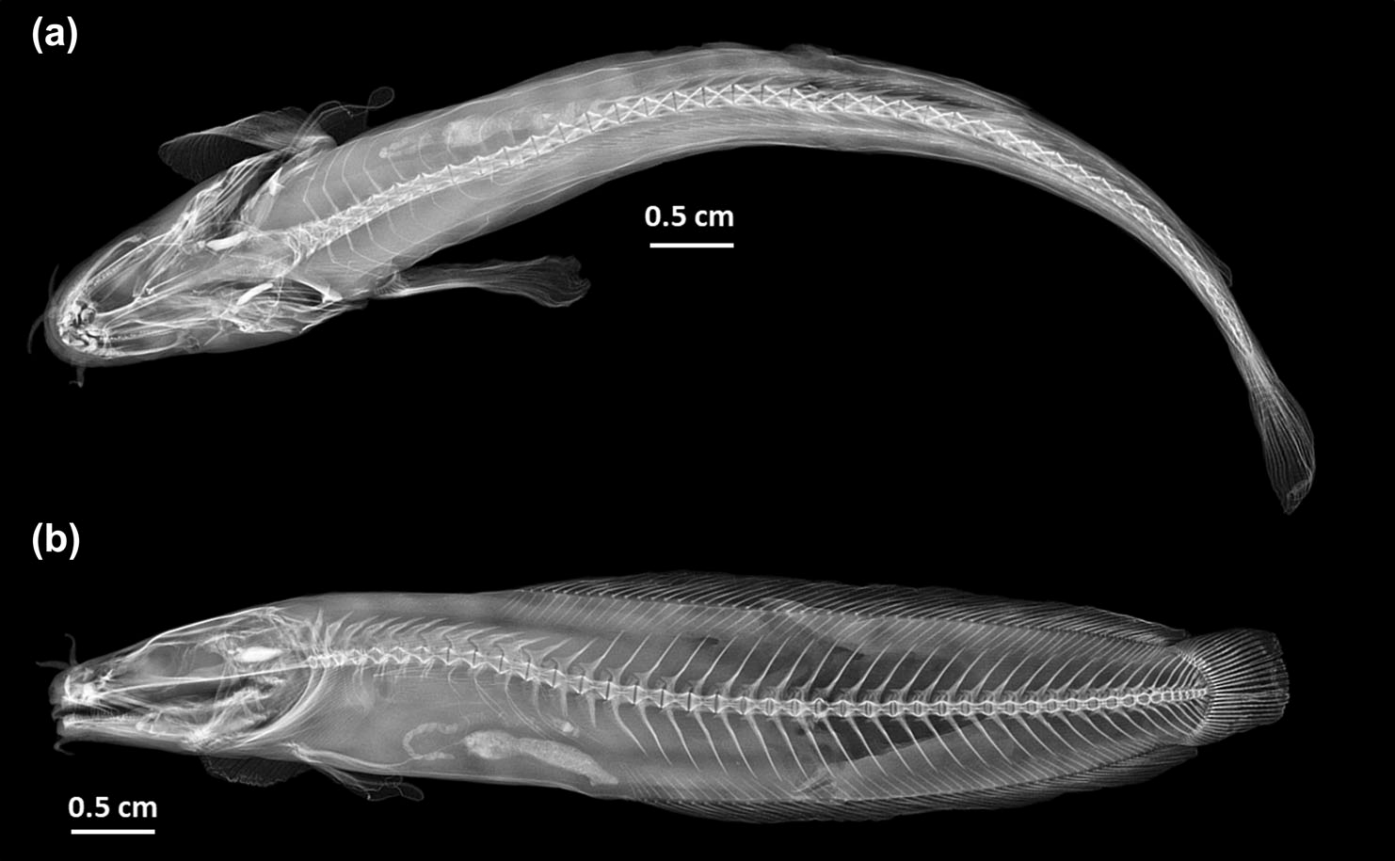
X‐ray footage of the holotype of *Gaidropsarus mauritanicus* sp. nov. (a) Dorsal view. (b) Lateral view.

**FIGURE 3:**
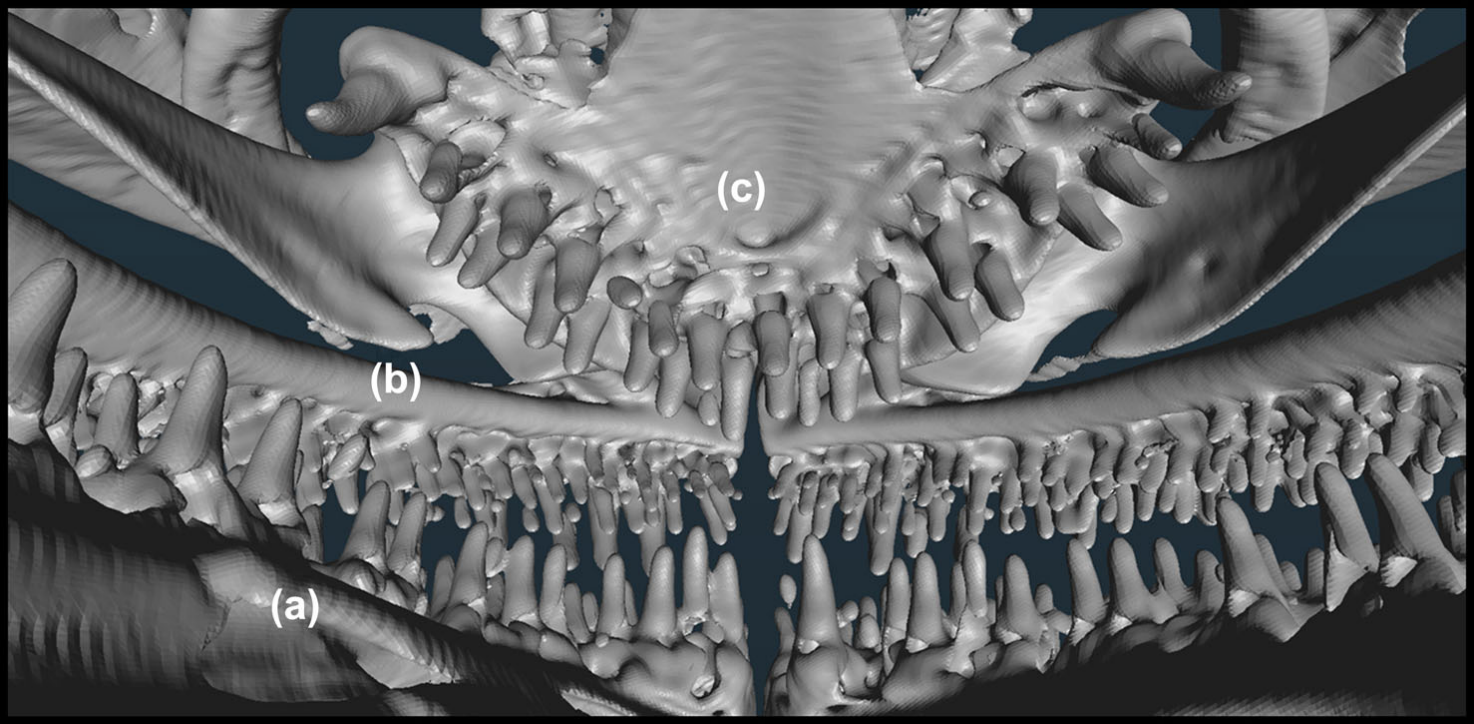
Three‐dimensional (3D) visualization of head based on micro‐CT scan showing the anterior dentition of *Gaidropsarus mauritanicus* sp. nov. (a) Mandibular teeth arranged in three rows. (b) Premaxillary teeth arranged in four rows. (c) V‐shaped vomer and its dentition.

**FIGURE 4:**
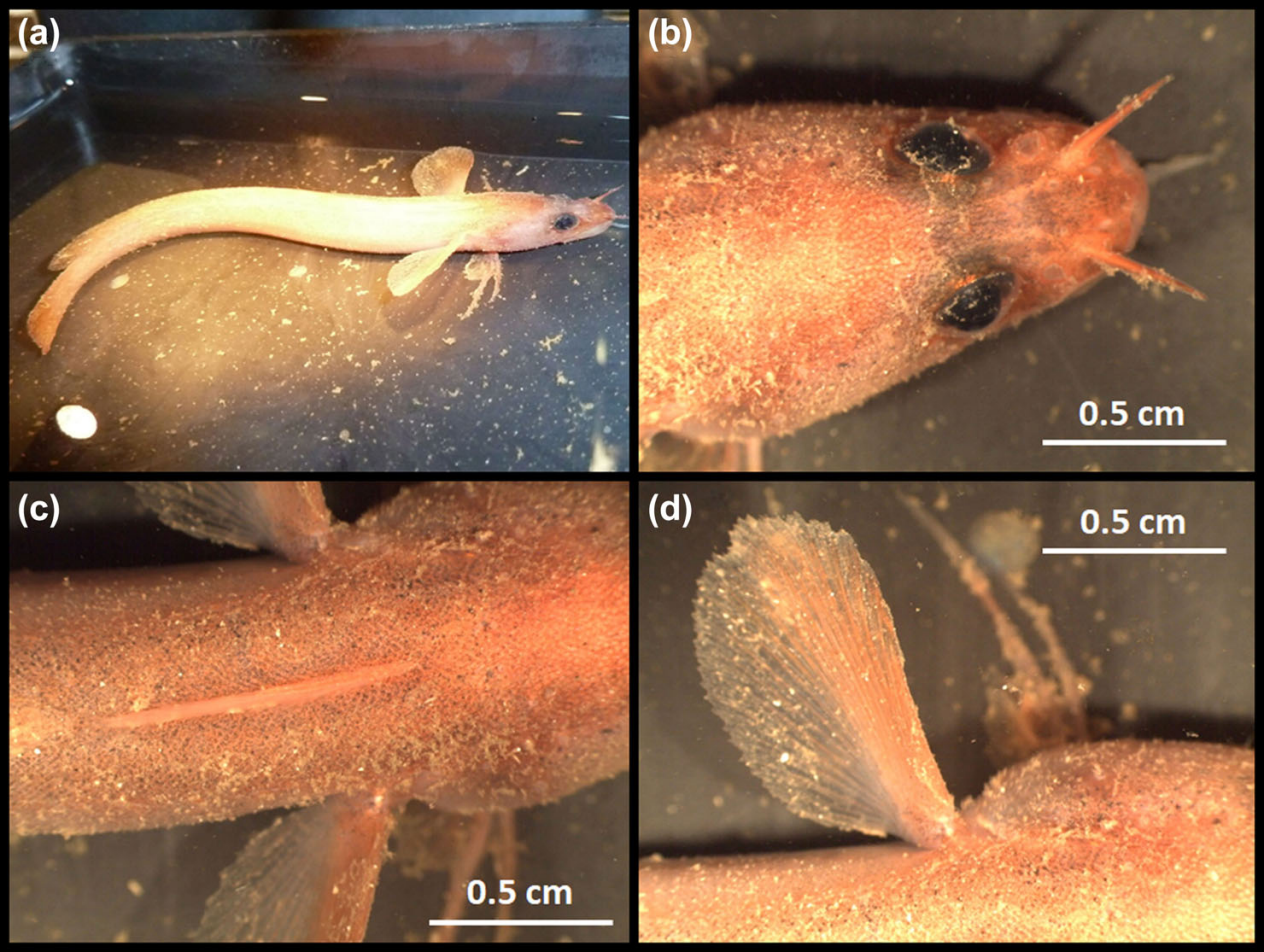
Photograph showing *in vivo* coloration of the holotype of *Gaidropsarus mauritanicus* sp. nov. in seawater after sampling. (a) Overview, dorso‐lateral side. (b–d) Details, dorsal view: (b) cephalic region, (c) first and second dorsal fins, and (d) left pectoral fin.

**FIGURE 5:**
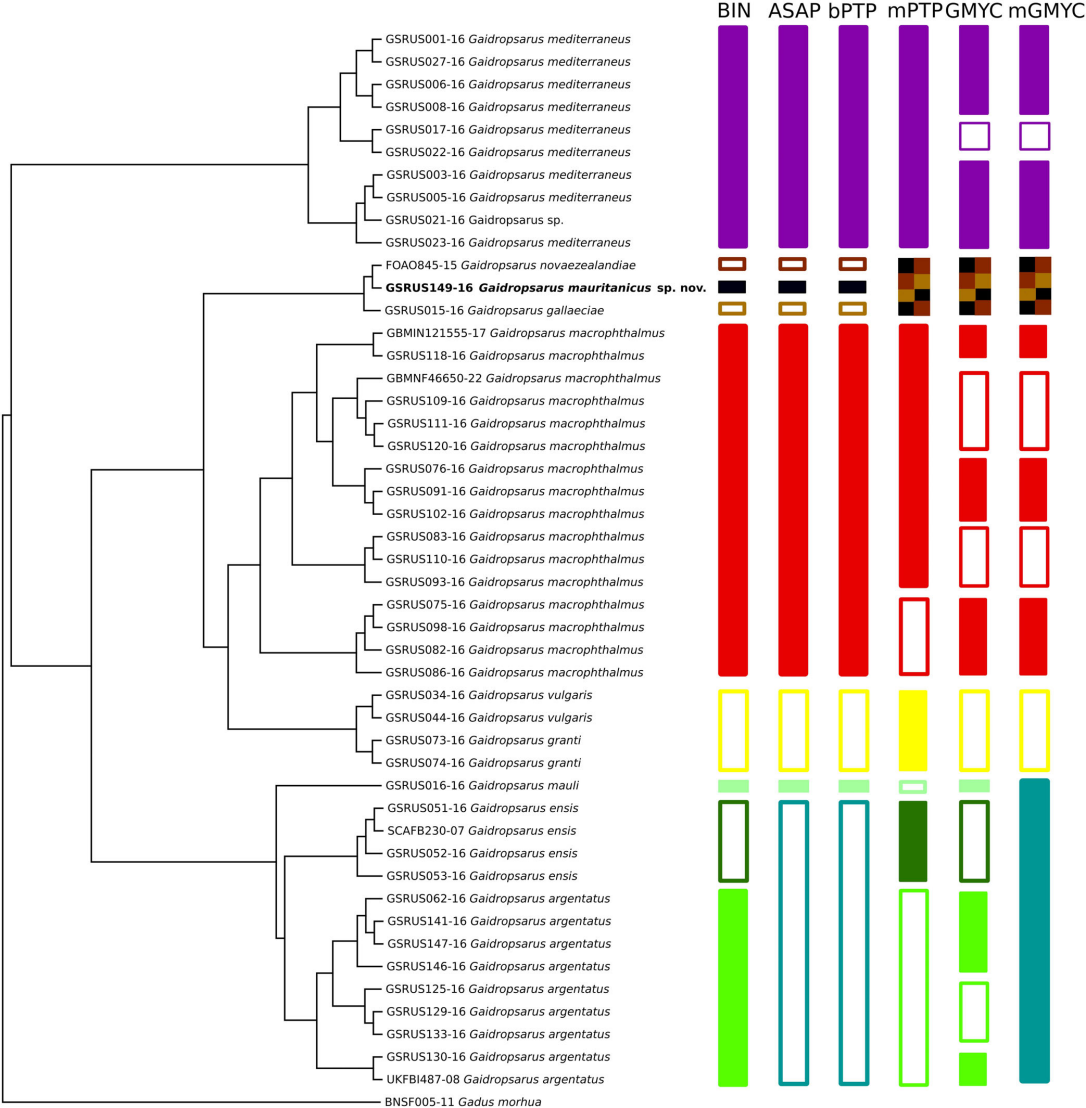
Species delimitation analysis of *Gaidropsarus mauritanicus* sp. nov., including distance‐based (Barcode Index Number [BIN] and the Assemble Species by Automatic Partitioning [ASAP]) and tree‐based (Bayesian Poisson Tree Processes [bPTP], multi‐rate Poisson Tree Processes [mPTP], Generalized Mixed Yule‐Coalescent [GMYC] and multiple threshold Generalized Mixed Yule‐Coalescent [mGMYC] ) methods.

**FIGURE 6:**
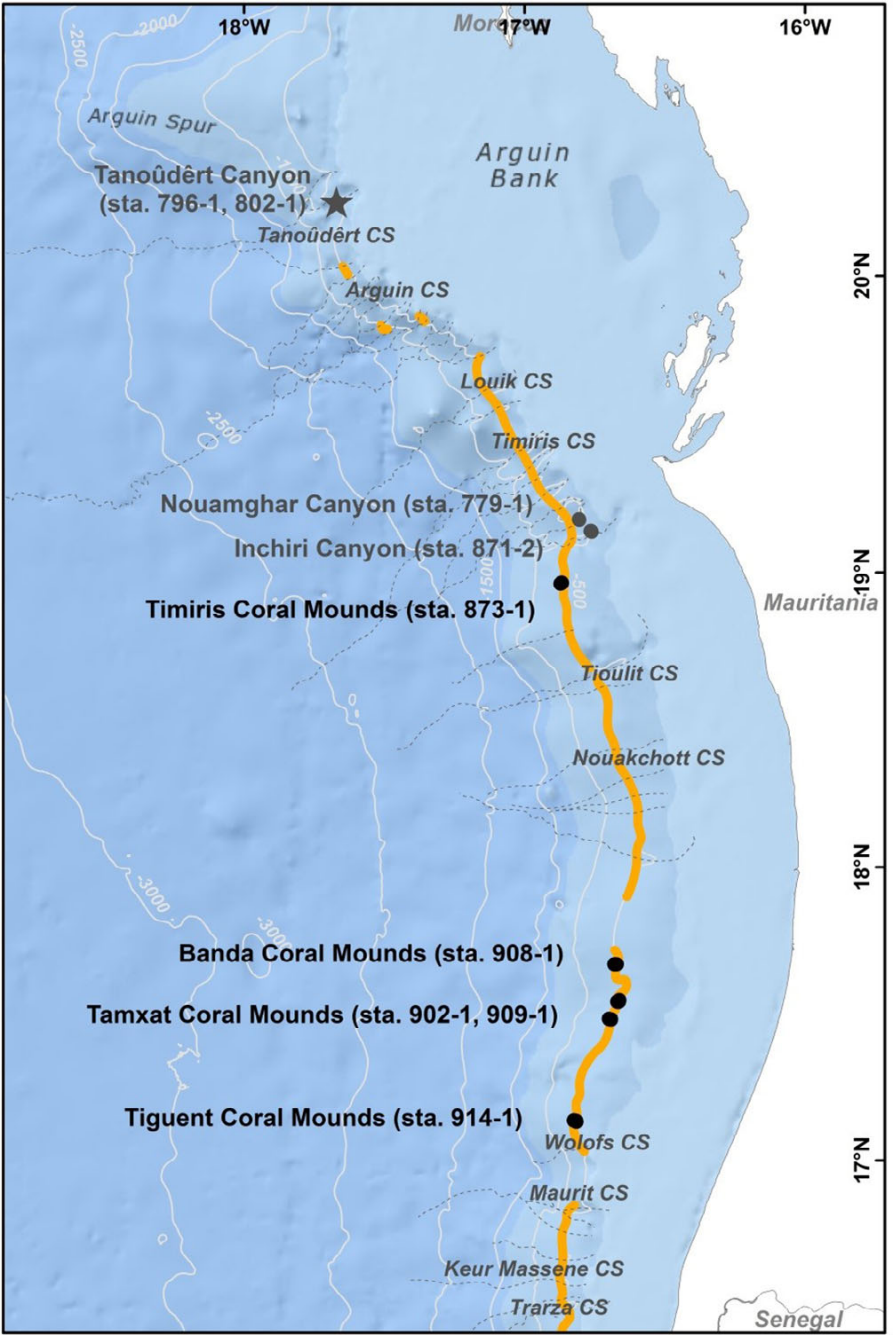
Biogeographical distribution of *Gaidropsarus mauritanicus* sp. nov. along the Mauritanian slope based on MSM 16/3 cruise material with main canyon systems (CS) and habitat‐forming deep‐water coral distribution (orange). Sampling station of the holotype (asterisk) and observations (dots) during remotely operated vehicle (ROV) dives with site name and GeoB 14***‐* station (=sta). Gray = canyons; black = coral mounds. Basemap from ESRI (2019), contours from GEBCO Compilation Group (2019), scleractinian distribution, canyon position, and names from Sanz et al. (2017).

**FIGURE 7:**
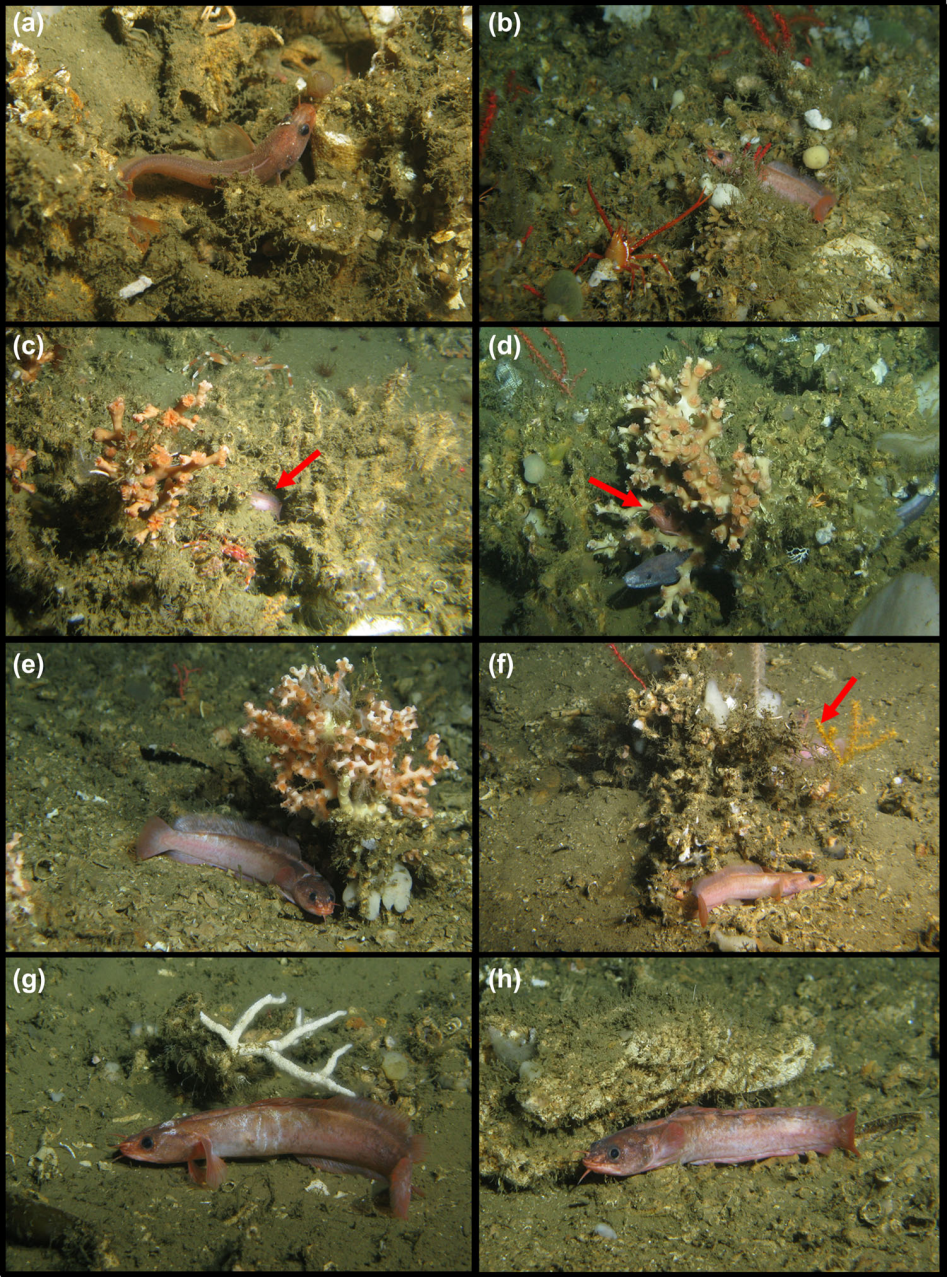
Representative remotely operated vehicle (ROV) images of *Gaidropsarus mauritanicus* sp. nov. in its habitat (copyright Tomas Lundälv from the Sven Lovén Centre for Marine Infrastructure of the University of Gothenburg, Sweden). (a) A small individual—similar in size to holotype—between coral rubble (Banda Coral Mounds, 523‐m water depth). (b) Inside a coral garden (*Swiftia phaeton*), sheltered between dead coral framework fragments and rubble (Timiris Coral Mounds, 492‐m water depth). (c) Hiding inside a dead coral framework, see red arrow (Tanoûdêrt Canyon, 610‐m water depth). (d) Coexistence with cf. *Japonoconger africanus* inside live *Desmophyllum pertusum* framework (red arrow) (Tamxat Coral Mounds, 501‐m water depth). (e) Below protective “canopy” of live *Madrepora oculata* (Tiguent Coral Mounds, 418 m). (f) Two individuals next to a dead scleractinian framework, which is colonized by sponges and various octocorals, among *Swiftia phaeton* (Tamxat Coral Mounds, 535‐m water depth; see also Video S3). (g) Next to a framework fragment, which is colonized by a branched bryozoan (*Celleporina* cf. *lucida*); note the whitish marks on the skin (Tamxat Coral Mounds, 479‐m water depth). (h) Adult individual finds shelter next to hardground (geology term) ledge; note the slightly varying coloration with respect to young individuals (Tiguent Coral Mounds, 434‐m water depth).

**FIGURE 8:**
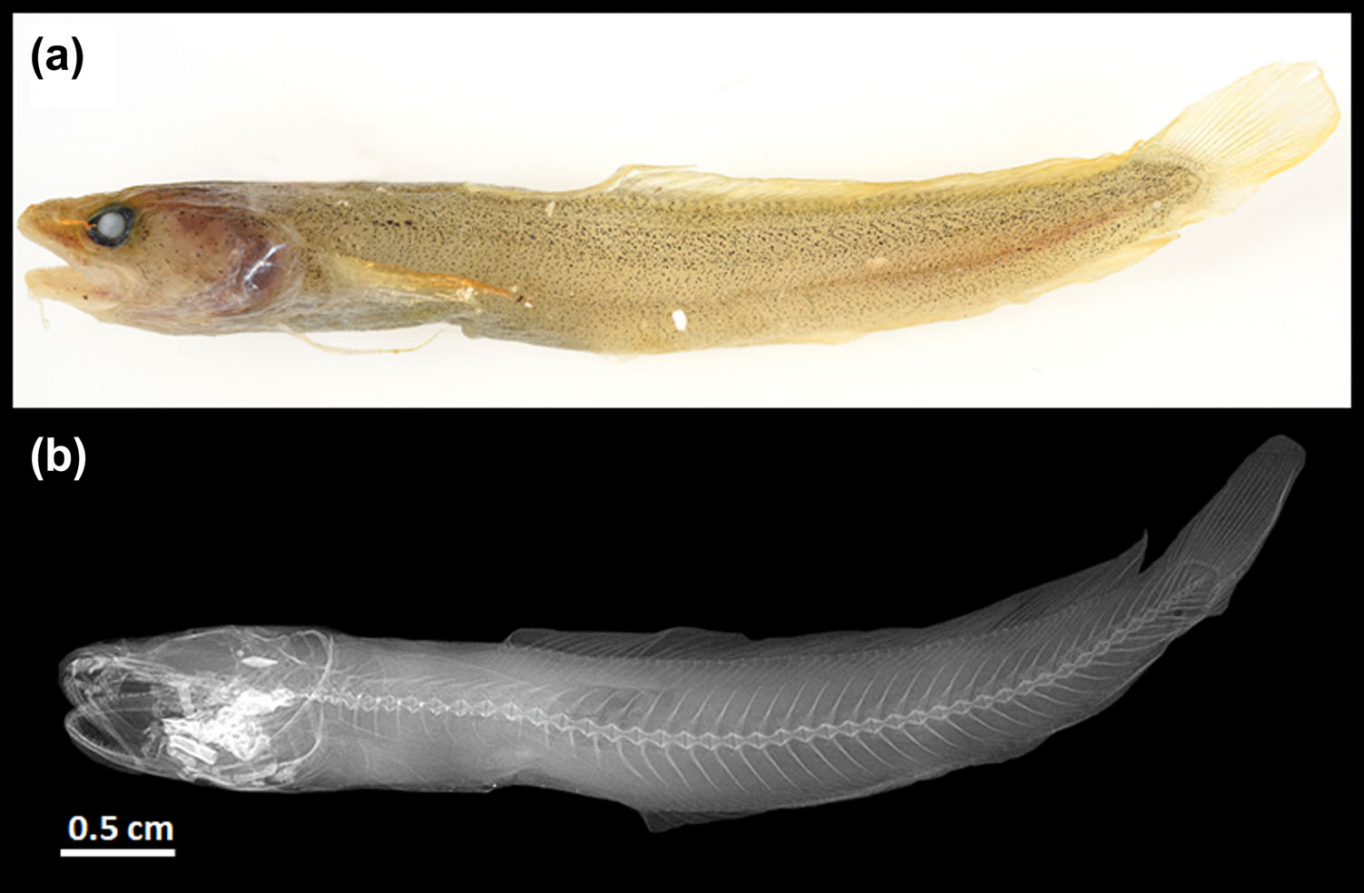
*Gaidropsarus* sp. from the Huon Marine Park, Tasmania (sampling location: 43° 58′ S, 147° 32′ E; process ID: FOAO845‐15, collection ID: CISRO H 7737‐01). (a) Photography of the collected individual after it was frozen. Taken by Carlie Devine (CSIRO Australian National Fish Collection). (b) X‐ray photography taken by John Pogonoski (CSIRO Australian National Fish Collection).

**FIGURE 9:**
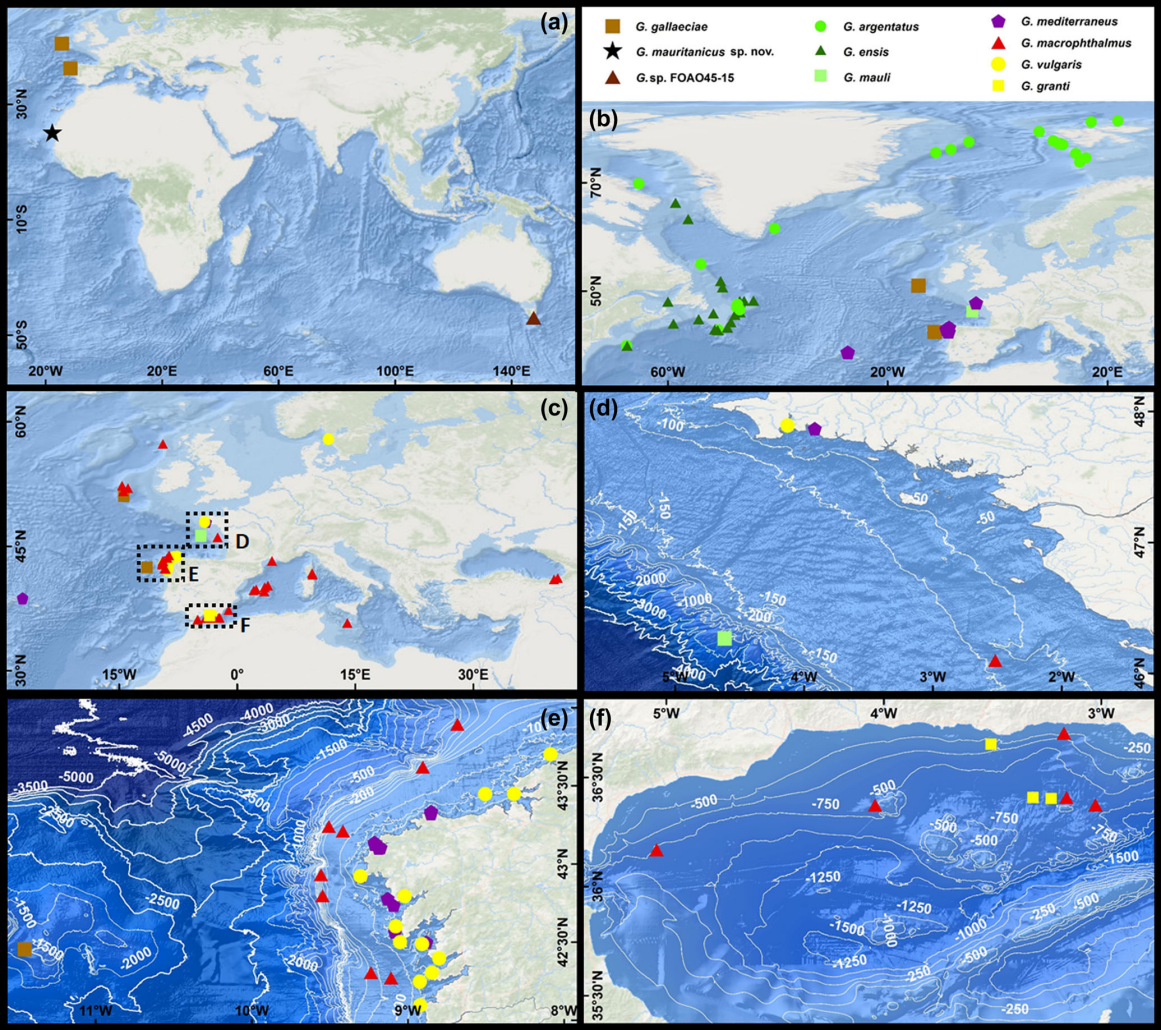
Geographical distributions of the genetically verified *Gaidropsarus* species records mined from BOLD‐System and GenBank. (a) Map with *Gaidropsarus mauritanicus* sp. nov. and its two closest related species (compare with Figure 5). (b) Distribution of boreal species. (c) Overview of records from the northeast Atlantic, Mediterranean Sea, and Black Sea; dashed boxes indicate close‐ups shown in d–f. *Gaidropsarus* spp. distribution (d) in the northern Bay of Biscay. (e) Off Galicia. (f) In the Alboran Sea. (a–c) Basemap from ESRI (2019). (d–f) Bathymetry and conducted contours based on EMODnet Digital Bathymetry (DTM 2022).

**FIGURE 10:**
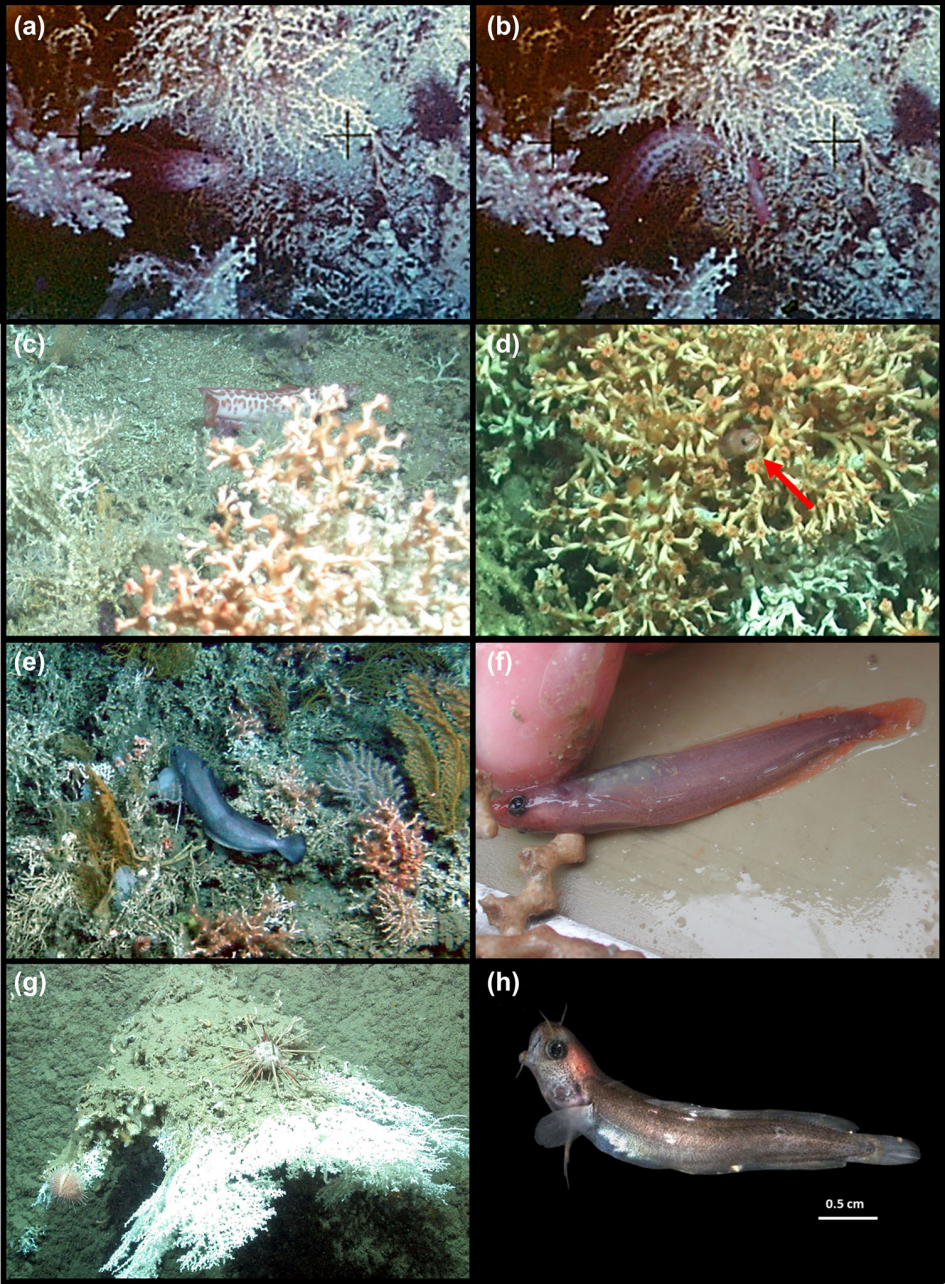
Members of the genus *Gaidropsarus* associated with framework‐forming deep‐water coral ecosystems. (a, b) *Gaidropsarus* sp. in 864‐m water depth on Galway Mound (copyright O. Pfannkuche and P. Linke 2004; see also Hebbeln et al., 2004 and Beuck, 2008); (c) *Gaidropsarus* cf. *granti* between live deep‐water coral framework on Galway Mound (copyright MARUM—Center for Marine Environmental Sciences, University of Bremen 2004); (d) *Gaidropsarus* sp. in a live *Desmophyllum pertusum* colony at Connacht Mound (copyright MARUM—Center for Marine Environmental Sciences, University of Bremen 2004); (e) Live deep‐water coral ecosystem at Thérèse Mound; image showing dense framework coverage and another gadiform fish (*Phycis blennoides*) (Copyright: Ifremer, Caracole cruise [2001]—https://doi.org/10.17600/1010080); (f) Juvenile *Gaidropsarus* cf. *gallaeciae* collected using giant box corer from 860‐m water depth at Thérèse Mound together with *Madrepora oculata* (Poseidon cruise 292, station 633‐1, 51°27.10′ N 11°44.98′ W); (g) Downward‐growing *M. oculata* colony collected during the M70/1 cruise from a steep cliff in the Bari Canyon, Mediterranean Sea (copyright MARUM—Center for Marine Environmental Sciences, University of Bremen 2006); (h) *Gaidropsarus* sp. individual found in the interstice of the coral framework, shown in (g) (see also Le Guilloux, 2011).

### 4.4 Dentition

Lanceolate and pointy teeth, pointing slightly backward. Teeth are present on the premaxilla, mandible, and vomer, as well as on the cerato‐ and pharyngobranchial tooth plates. Premaxillary teeth are smaller than dentary teeth. The dentition in the upper and lower jaws consists of densely arranged tooth bands. Premaxillary teeth are arranged in four rows. Mandibular teeth are arranged in three rows, where the teeth of the second row are slightly larger than the other. V‐shaped vomer possesses two rows of teeth. The most distal tooth is strongly pronounced and canine like. Pharyngeal teeth are arranged in several rows. The pharyngeal teeth become larger moving from the outside inward. Gill rakers are present as dentated tubercles along the first branchial arch. There are seven to eight gill rakers on the outer row of the branchial arch.

### 4.5 Coloration

The characterization of the *in vivo* coloration of *G. mauritanicus* sp. nov. is based on the living holotype and additional ROV footage documenting various individuals from deep‐water coral ecosystems off Mauritania. Living individuals exhibit a predominantly pinkish hue, with the dorsal side displaying a darker brownish shade than the ventral side, which appears brighter and more pinkish than the lateral side. Older individuals have several pale blotches along the base of the dorsal fins (Figure 7e,h). Younger individuals do not possess these blotches (Figures 4 and 7a). Notably, the pores of the head lateral‐line system exhibit a brighter coloration in contrast to the rest of the head. Conversely, the holotype's coloration, preserved in ethanol (see also Figure 1), appears opaque, with scattered melanophores sparsely distributed throughout the body. The iris is dark, and the pupil appears turbid and whitish. The fins exhibit opacity at the base and gradually transition to translucency toward the distal ends. No discernible patterns are evident.

### 4.6 Etymology

The species name “*mauritanicus*” is derived from the Latin name of the Islamic Republic of Mauritania, known for its species‐rich marine ecosystems, among them the most extensive known “chain”‐shaped, habitat‐forming deep‐water coral ecosystem, to which this species is associated.

### 4.7 Comparison with other species

Table 2 presents a comprehensive overview of the morphometric characteristics of *G. mauritanicus* sp. nov., highlighting the key distinctions observed compared to all other species within the genus *Gaidropsarus*, as documented by Bañón et al. (2022). Table 3 additionally incorporates supplementary observations gathered from the study conducted by Biscoito and Saldanha (2018) and Bañón et al. (2022).

**TABLE 2:** Comparison of the morphometric and meristic characteristics of *Gaidropsarus mauritanicus* sp. nov. with all 13 valid *Gaidropsarus* species from Bañón et al. (2022) and Biscoito and Saldanha (2018).

	*Gaidropsarus mauritanicus* sp. nov.	*Gaidropsarus argentatus*	*Gaidropsarus capensis*	*Gaidropsarus ensis*	*Gaidropsarus gallaeciae*
As % SL					
Head length	25.8	19.7–25.1	19.4–22.5	19–22	21.1–25.2
First predorsal length	22.8	20.7–22.6	‐	18.7–20.2	21.8–27.7
Third predorsal length	39.8	31.7–36.8	‐	29.1–32.3	33.7–40.4
Second dorsal‐fin base length	16.9	8.6–11.4	12.2–13.2	8–11.3	9.8–11.7
Third dorsal‐fin base length	56.4	57.1–62.4	‐	59.3–64.4	55.9–64.4
Anal‐fin base length	48.8	38.7–39.8	48.4–49	39.9–46.3	39.6–48
Pectoral‐fin length	13.8	16.1–18.9	‐	17–20	15.3–17.5
Pelvic‐fin length	35.7	18.1–21.5	‐	17–26.3	16.2–19
Pre‐anal length	47.6	51.4–53.4	‐	48–50	43.4–49.1
Body depth	9.8	15.6–23.5	16.5–17.3	16.7–25.2	15.7–21.6
Prepectoral length	29.2	20.7–28	‐	17.8–21.3	22–27.5
Prepelvic length	21.3	16.9–19.9	‐	12.7–16	16.8–21.3
Caudal peduncle height	6.2	5.5–7.4	7–8.1	5.1–7.2	6.2–7.7
As % HL					
Snout length	25.4	25.2–27	28.2–33.2	23.6–27.9	20.9–25
Eye diameter	38.1	14.8–21.8	16.1–20.9	17.3–24.5	15.8–20.5
Postorbital length	50.8	57.3–58.4	‐	54.1–59.1	54.7–64.3
Interorbital space	19.0	13.1–23.1	13.5–19.5	14.4–25.1	21.7–28
Upper jaw length	37.3	44.7–47.7	48.8–52.1	45.3–64.8	37.9–47.2
Lower jaw length	35.6	36.6–41.1	‐	36.1–60.3	32–40.1
Chin barbel length	20.0	19.8–23.8	‐	15.1–20.8	16.7–22.6
First dorsal‐fin ray length	24.9	24.1–43	19.5–32.5	82.1–145.5	15.8–27
Meristics					
Third dorsal‐fin rays	57	52–65	43–52	52–64	54–60
Anal‐fin rays	53	43–51	37–43	40–48	44–52
Pectoral‐fin rays	24	22–25	18–21	20–27	21–23
Pelvic‐fin rays	7	7–8	6–7	6–7	7
Gill rakers outer row	7–8	8–11	4–9	11–13	7–9
Gill rakers inner row	‐	10–11	8–9	12–14	6–9
Vertebrae	44	49–53	41–43	50–54	43–44
Head lateral‐line system					
SO canals	4	4	4	4	‐
IO canals	10	12–13	11	11–12	‐
STC canals	1	2	3	2	‐
POM canals	14	11–13	13	13–14	‐

	*Gaidropsarus granti*	*Gaidropsarus insularum*	*Gaidropsarus macrophthalmus*	*Gaidropsarus mauli*	*Gaidropsarus mediterraneus*
As % SL					
Head length	20.9–25.5	18.7–21.5	19.3–23.2	23.4–25.4	18.8–24
First predorsal length	‐	‐	20.5–22.9	22.8–23.9	18.6–18.9
Third predorsal length	21.1–37.9	‐	33.3–38.3	36–36.3	20.1–38.5
Second dorsal‐fin base length	10.7	8.5–9.8	8.6–11.7	‐	13.2–18.4
Third dorsal‐fin base length	54.4–59.7	65.1–67.5	55.6–66.3	‐	54.1–60.9
Anal‐fin base length	43.6–45.6	46.4–49.6	48.5–50	‐	45–52.2
Pectoral‐fin length	13.8–15.4	‐	14.7–15.5	17.8–19.4	12.3–14.6
Pelvic‐fin length	15.5–23.1	‐	9.6–16.1	27.5–33.3	13–15.5
Pre‐anal length	48.7–54.8	‐	44–47.6	50.8–53.1	44.1–51.1
Body depth	13.1–14	‐	14.2–19.5	15.2–21.9	14–19.3
Prepectoral length	‐	‐	19.3–25	‐	20.5–22.7
Prepelvic length	‐	‐	16.3–20.2	‐	15–17.2
Caudal peduncle height	5.6–6.9	6.8–8.5	4.8–7.1	5.6–6.8	4.5–6.3
As % HL					
Snout length	19.3–29.2	27.6	21–26	25.4–26.7	18.8–30.4
Eye diameter	13.7–18.8	‐	16–23.7	10.4–12	13.3–22.5
Postorbital length	59.6	‐	54.3–59.1	‐	60.63.5
Interorbital space	10.5–17.6	16.7–19.4	12.5–26.5	20.9–21.3	9.1–25.7
Upper jaw length	42.9	59.2–61.3	46.2–52.9	‐	42.9–45.6
Lower jaw length	41.7	48.4–55.1	36.6–44	‐	38.9–40.2
Chin barbel length	‐	‐	14–22.2	26.9	15.3–18.5
First dorsal‐fin ray length	12.7–14.9	11.2–25	10.1–25.1	21.3–25.4	14.9–42
Meristics					
Third dorsal‐fin rays	55–60	66–70	48–59	57–58	48–63
Anal‐fin rays	45–52	50–57	40–50	46–47	41–53
Pectoral‐fin rays	20–22	19–22	17–22	25–26	15–19
Pelvic‐fin rays	7–8	‐	6–7	9	5–8
Gill rakers outer row	10	7	6–9	1 + 7	7–10
Gill rakers inner row	‐	9	8–11	1 + 8	9–11
Vertebrae	44–47	47–49	43–47	47–48	46–50
Head lateral‐line system					
SO canals	‐	‐	4	4	4
IO canals	‐	‐	12–13	12	12–13
STC canals	‐	‐	3	2	3 on each side
POM canals	‐	‐	13	11–13	13

	*Gaidropsarus novaezealandiae*	*Gaidropsarus pakhorukovi*	*Gaidropsarus parini*	*Gaidropsarus vulgaris*
As % SL				
Head length	17.9–20.7	23.7–24.7	22.1–22.8	23.6–25.9
First predorsal length	‐	24.4–25.3	17.8–18.5	22.1–24
Third predorsal length	‐	‐	‐	36.4–38.1
Second dorsal‐fin base length	‐	12.8–15.5	10.4–11.6	11.3–13.9
Third dorsal‐fin base length	58.5–65.3	55.3	56–58.1	54.9–61.1
Anal‐fin base length	48.2–51.5	41.7	43.8–48.6	40.5–45.3
Pectoral‐fin length	‐	17.7–19.2	17.3–17.8	14.1–15.4
Pelvic‐fin length	‐	‐	19.9–20.7	17.4–20.3
Pre‐anal length	‐	‐	45.2–48.5	48.9–54.8
Body depth	‐	‐	‐	14.8–20.4
Prepectoral length	‐	‐	‐	23.5–25.4
Prepelvic length	‐	22.4	‐	18.6–20.7
Caudal peduncle height	6.3–8.1	6.5	6.7–7.1	7–8.5
As % HL				
Snout length	‐	‐	‐	21.2–26.6
Eye diameter	15.2–19	17.2–19.8	13.9–16.4	10.5–16.7
Postorbital length	‐	‐	‐	55.3–65.2
Interorbital space	15.2–18.7	16	‐	14.4–19.5
Upper jaw length	‐	‐	‐	42.3–49.3
Lower jaw length	‐	‐	‐	36.8–40
Chin barbel length	‐	‐	‐	19.6–24.2
First dorsal‐fin ray length	20–27.9	12–15.1	26.7–28	9.5–16.9
Meristics				
Third dorsal‐fin rays	56–69	60–62	60–64	56–64
Anal‐fin rays	50–59	50–51	52–53	46–54
Pectoral‐fin rays	20–21	22–26	23–25	20–24
Pelvic‐fin rays	7–8 (5)	7–8	7–8	6–7
Gill rakers’ outer row	6–8	9	7	7–9
Gill rakers’ inner row	9–10	9	10	10–11
Vertebrae	46–49	46–47	47–48	46–49
Head lateral‐line system				
SO canals	4	4	4	4
IO canals	11–13	12	12–13	12–13
STC canals	3	‐	3	3
POM canals	13–14	13	13	13

Abbreviations: HL, head length; SL, standard length, IO, Infraorbital canal; SO, Subraorbital canal; STC, Supratemporal canal; POM, Preoperculo‐mandibular canal.

**TABLE 3:** Notable morphological characteristics and known geographical and bathymetrical distribution of *Gaidropsarus mauritanicus* sp. nov. compared to the valid species records of the genus *Gaidropsarus* following Bañón et al. (2022) and Biscoito and Saldanha (2018) indicated by *; depth range information was gathered from Fish Base (Froese & Pauly, 2023) indicated by **. Biogeographical distribution of genetically validated *Gaidropsarus* species with world seas names from Flanders Marine Institute (2018), depth information from GEBCO (2023) with records >0 listed as 0, exact depth mentioned in metadata from samples in brackets; Province (ecoregion[s]) after Spalding et al. (2007).

Species	*Differentiating characteristics from *Gaidropsarus mauritanicus* sp. nov.	*Geographical distribution and **depth [m]	World seas and GEBCO depth [m]	Province (ecoregion[s])
*G. mauritanicus* sp. nov.	Large eyes (38.1.% HL), long pelvic fin (35.7% SL), low number of vertebrae (44)	Eastern central Atlantic 595	Eastern North Atlantic Ocean 622 (595)	West African Transition (Sahelian Upwelling)
It differs from other valid *Gaidropsarus* species by having:				
*Gaidropsarus argentatus*	Longer second dorsal fin (16.9% SL vs. 8.6%–11.4% SL), longer anal fin (48.8% SL vs. 38.7%–39.8% SL), longer pelvic fin (35.7% SL vs. 18.1%–21.5% SL), fewer vertebrae (44 vs. 49–53), smaller upper jaw (37.3% HL vs. 44.7%–47.7% HL)	North Atlantic, off Newfoundland and Labrador and west of British Isles 150–2260	Arctic Ocean, Greenland Sea, northern Norwegian Sea, western North Atlantic Ocean, Labrador Sea, northwestern Davis Strait 233–2158	Arctic (western part of North Sea and East Barents Sea, eastern part of North Greenland, southern part of East Greenland Shelf, Baffin Bay—Davis Strait, northern Grand Banks—southern Labrador), cold‐temperate Northwest Atlantic (southern Grand Banks—south Newfoundland, Gulf of Maine/Bay of Fundy)
*Gaidropsarus capensis*	Larger head (25.8% SL vs. 21.5% SL), more third dorsal‐fin rays (57 vs. 43–52), more anal‐fin rays (53 vs. 37–43), and fewer pectoral‐fin rays (24 vs. 18–21)	Southeastern Atlantic and Southwestern Indian Ocean From tide pools to 50	‐	‐
*Gaidropsarus ensis*	Greater head length (25.8% of vs. 19.7%–22.5% HL), shorter pectoral fin (13.8% SL vs. 17%–20% SL), longer pelvic fin (35.7% SL vs. 17–26.3% SL), more anal‐fin rays (53 vs. 40–48), fewer vertebrae (44 vs. 50–54)	North Atlantic, off Newfoundland and Labrador 0–2000	Western North Atlantic Ocean, Labrador Sea, mid Davis Strait 83–2875	Cold‐temperate Northwest Atlantic (southern Grand Banks—south Newfoundland, Gulf of St. Lawrence—eastern Scotian Shelf, Scotian Shelf, Gulf of Maine/Bay of Fundy), Arctic (northern Grand Banks—southern Labrador)
*Gaidropsarus gallaeciae*	Longer pelvic fin (35.7% SL vs. 16.2%–19% SL), smaller interorbital distance (19.0% HL vs. 21.7%–28% HL), different geographical distribution (Although both species are closely related within the genus *Gaidropsarus*, their cytochrome oxidase 1 units differ by 4.6% [following Barros‐Garcia et al., 2018], providing support for *G. mauritanicus* sp. nov. as a distinct species.)	Northeastern Atlantic, Galicia Bank, and Porcupine Bank 751–788	Eastern North Atlantic Ocean 767–1592	Northern European Seas (Celtic Seas), Lusitanian (South European Atlantic Shelf)
*Gaidropsarus granti*	Longer second dorsal fin (16.9% SL vs. 10.7% SL), longer pelvic fin (35.7% SL–15.5%–23.1% SL), more pectoral‐fin rays (24 vs. 20–22)	Southwestern Atlantic, in Porcupine Bank (southwest of Ireland), Galicia Bank, Azores, Madeira and Canary islands, and Mediterranean 20–823	Alboran Sea 316–930	Mediterranean Sea (Alboran Sea)
*Gaidropsarus insularum*	Longer head (25.8% SL vs. 18.7%–21.5% SL), longer second dorsal fin (16.9% SL vs. 8.5%–9.8% SL), shorter third dorsal fin (56.4% SL vs. 65.1%–67.5% SL)	Southeastern Atlantic and Southwestern Indian Ocean Littoral (tide pools)	‐	‐
*Gaidropsarus macrophthalmus*	Longer second dorsal fin (16.9% SL vs. 8.6%–11.7% SL), longer pelvic fin (35.7% SL vs. 9.6%–16.1% SL), smaller upper jaw (37.3% HL vs. 46.2%–52.9% HL), more pectoral‐fin rays (24 vs. 22), fewer vertebrae (44 vs. 45–47)	Northeastern Atlantic from Faroe Islands and British Isles to south of the Azores and Mediterranean 150–600	Bay of Biscay, eastern North Atlantic Ocean, Alboran Sea, Mediterranean Sea—Western Basin, Balearic Sea, Tyrrhenian Sea, Mediterranean Sea—Eastern Basin, Black Sea 40–2401	Northern European Seas (Celtic Seas), Lusitanian (Azores Canaries Madeira, South European Atlantic Shelf), Mediterranean Sea (Ionian Sea, Western Mediterranean, Alboran Sea), Black Sea
*Gaidropsarus mauli*	Smaller pectoral length (13.8% SL vs. 17.8%–19.4% SL), greater eye diameter (38.1% HL vs. 10.4%–12% HL), higher number of anal‐fin rays (53 vs. 46–47), fewer pelvic‐fin rays (7 vs. 9), lower number of vertebrae (44 vs. 47–48)	Atlantic, Azores, and Bay of Biscay 870–1500	Bay of Biscay 1229	Lusitanian (South European Atlantic Shelf)
*Gaidropsarus mediterraneus*	Longer pelvic fin (35.7% of SL vs. 13%–15.5% of SL), shallower body depth (9.8% SL vs. 14%–19.3% SL), more pectoral‐fin rays (24 vs. 15–19), and fewer vertebrae (44 vs. 46–50)	Northeastern Atlantic, from Norway and British Isles south to Morocco, including Canaries, Azores, and Madeira, Mediterranean Sea, and Black Sea 1–450	Eastern North Atlantic Ocean, Bay of Biscay 0–24	Lusitanian (Azores Canaries Madeira, South European Atlantic Shelf)
*Gaidropsarus novaezealandiae*	Greater head length (25.8% SL vs. 17.9%–20.7% SL), lower number of vertebrae (44 vs. 46–49)	Southwestern Pacific, New Zealand and south of Tasmania 0–50, but two specimens collected at 300–500	‐	‐
*Gaidropsarus pakhorukovi*	Shorter pectoral fin (13.8% SL vs. 17.7%–19.2% SL) and fewer third dorsal‐fin rays (57 vs. 60–62)	Southwestern Atlantic, Rio Grande Seamount ?–690	‐	‐
*Gaidropsarus parini*	Shorter pectoral fin (13.8% SL vs. 17.3%–17.8% SL), fewer third dorsal‐fin rays (57 vs. 60–64), and fewer vertebrae (44 vs. 47–48)	Southeastern Pacific, Nazca Ridge ?–310	‐	‐
*Gaidropsarus vulgaris*	Longer pelvic fin (35.7% SL vs. 17.4%–20.3% SL), shorter upper jaw (37.3% HL vs. 42.3%–49.3% HL), and fewer vertebrae (44 vs. 46–49)	Northeastern Atlantic, from Norway and Iceland south to Gibraltar, including Madeira and Mediterranean 10–120	Eastern North Atlantic Ocean, Bay of Biscay, Kattegat 0–100	Lusitanian (South European Atlantic Shelf), Northern European Seas (North Sea)

### 4.8 Genetic species delimitation analysis

The validity of *G. mauritanicus* sp. nov. has been tested through several DNA‐based species delimitation analyses (Figure 5). Both distance‐based methods (Barcode Index Number [BIN] and the Assemble Species by Automatic Partitioning [ASAP]) agreed to considering the sequence GSRUS149‐16 *G. mauritanicus* sp. nov. as an independent Molecular Operational Taxonomic Unit. Among the tree‐based methods, only bPTP showed similar results, whereas mPTP, GMYC, and mGMYC clustered GSRUS149‐16 together with *G. gallaeciae* (GSRUS015‐16) and a sequence of unknown species affiliation (FOAO845‐15) from Tasmania (Figures 5 and 8).

### 4.9 Biogeographical distribution, habitat, and accompanying fauna

The holotype derives from the Tanoûdêrt Canyon, Mauritania, collected in 595‐m water depth using grab sampling. The species was additionally observed thrice during the ROV dive carried out in this canyon (GeoB 14796‐1). The Tanoûdêrt Canyon was the northernmost canyon studied during the MSM 16/3 cruise. The species was further observed during dives in the Timiris Canyon System—particularly in the Nouamghar (one individual) and Inchiri Canyon (two individuals). Further south, individuals were documented during ROV dives investigating different coral mounds along the “coral mound chain,” namely Timiris (six individuals), Banda (one individual), Tamxat (five individuals), and Tiguent Coral Mounds (four individuals; see Figure 6). The deepest record was observed at 613 m in the Tanoûdêrt Canyon, the shallowest at 416 m on the Tiguent Coral Mounds. The temperature range of the 22 presumably *G. mauritanicus* sp. nov. individuals observed was between 9.67°C (Nouamghar Canyon) and 11.58°C (Tiguent Coral Mounds), and oxygen values were between 1.64 mL/L (Tanoûdêrt Canyon) and 1.12 mL/L (Tiguent Coral Mounds).

The holotype was collected together with a diverse assemblage of live deep‐water fauna, including corals, such as *Desmophyllum pertusum*, *Desmophyllum dianthus*, *Caryophyllia* sp., and *Swiftia phaeton*, as well as larger sponge colonies, bryozoans, ophiuroids, polychaetes, decapods, hydrozoans, gastropods, bivalves, and chitons with dead *D. pertusum* framework, coral rubble, and olive‐colored silt as substrate (Gil et al., 2020; Matsuyama et al., 2015; Sampaio et al., 2022). These findings indicate *G. mauritanicus* sp. nov. as a demersal species associated with deep‐water coral ecosystems. This is further supported by ROV footage deriving from the submarine canyons and coral mounds off Mauritania. Individuals exclusively occurred either on dense coral rubble or were associated with dispersed, patchy, or dense live and/or dead scleractinian framework.

The distribution of framework‐forming deep‐water coral ecosystems extends northwards off Western Sahara southwards off Senegal (Wienberg et al., 2023). It can be assumed that future studies will find this species along the slope north and south of Mauritanian waters.

### 4.10 Behavior

Individuals observed during ROV dives were mainly resting on the bottom (Video S3), preferentially associated with live or dead coral framework or framework portions (inside, below, or next to), or, in the absence of framework, next to larger protective structures like hard‐ground ledges (see Figure 7). When individuals felt threatened (e.g., by the approaching ROV), they attempted to hide in or below protective structures (see also Video S3). Observed swimming was fast paced in minimal altitude over ground and along protective structures (Video S2).

## 5 DISCUSSION

### 5.1 Morphological and genetic identification of *G. mauritanicus* sp. nov.

The number of vertebrae divides the genus *Gaidropsarus* into two clades (Bañón et al., 2022), with one clade possessing species with fewer than 45 vertebrae (*G. mauritanicus* sp. nov., *G. gallaeciae, G. macrophthalmus, G. capensis*, and *G. granti*) and the other clade with species having 45 or more vertebrae. The new species is further distinguished from all other species by its long pelvic fin, large eyes, and short pectoral fin. The pinkish coloration of *G. mauritanicus* sp. nov. is shared with *G. ensis, G. argentatus, G. mauli*, and *G. gallaeciae*, but adults of this new species differ from these others by having a brownish hue on the dorsum and several pale blotches along the base of the dorsal fins.

The DNA‐based delimitation analyses with a multi‐locus approach have already been discussed in Barros‐García et al. (2022). Discrepancies have been found; for instance, *COI* had failed to differentiate between the closely related *G. granti* and *G. vulgaris*, which are grouped together in all analyses. Similarly, three of the analyses clustered together *G. argentatus* and *G. ensis*, which are two valid independent species. These discrepancies highlight that single‐locus results must always be interpreted cautiously and integrated with other available data (e. g., morphology, ecology), testing multiple methods to assess congruence among them, a widely used approach in species delimitation analysis (Rannala, 2015).

Both the distance‐based methods (BIN and ASAP) agree with the morphospecies *G. mauritanicus* sp. nov., whereas three out of four tree‐based methods (mPTP, GMYC, and mGMYC) combined *G. mauritanicus* sp. nov. with *G. gallaeciae* and a third unknown individual (FOAO845‐15). Distance‐based approaches rely on a threshold value to determine whether a pair of specimens belong to the same species; values higher than optimal merge different species, whereas a lower value will split species into two or more (Ratnasingham & Hebert, 2013). A study comparing *COI* sequences representing more than 2000 animal species found that 98% of pair‐species comparisons showed divergence values over 2%, except cnidarians, and therefore proposed the 2% value as a threshold for delimitation for animal species (Hebert et al., 2003). The genetic distance between *G. mauritanicus* sp. nov. and the two most close sequences range from 4.76% to 4.92% (data not shown). It is not surprising, therefore, that BIN analysis clustered them independently because the threshold applied in BIN analyses is 2.2% (Ratnasingham & Hebert, 2013). Similarly, the threshold used in the selected partition of ASAP is 3.3%, which explains the similarity in the results. Even when the genetic distance between *G. mauritanicus* sp. nov. and *G. gallaeciae* is lower (4.92%) compared to the average genetic distance among *Gaidropsarus* species (around 10%–15%) (Barros‐García et al., 2018), it is adequate to be considered a separate species from a DNA‐barcoding standard (Hebert et al., 2003). Several hypotheses can explain the disparity observed in genetic distances among *Gaidropsarus* species, ranging from recent speciation to a past introgressive event, but more evidence should be obtained to test these theories.

Instead of a threshold, tree‐based approaches rely on the evolutionary history of the selected marker. However, this is a potentially problematic assumption if the gene does not reflect the species’ evolutionary history and speciation, especially when using a single‐locus approach (Sagorny et al., 2019). PTP uses the expected number of substitutions to estimate branch processes, but it is limited to two independent distributions (one for speciation and one for coalescence), which ignore variation among species due to different population sizes and demographic histories in contrast to the mPTP (Kapli et al., 2017). mPTP works under the same principles but allows more than two independent distributions through the tree. Nevertheless, mPTP can be less suitable for datasets with incomplete sampling, that is, cluster together taxa on long branches with no close relatives in the dataset (Servis et al., 2020).

In the present study, four putative species are represented for a single individual, including *G. mauritanicus* sp. nov., *G. gallaeciae*, and FOAO845‐15, which are clustered together by GMYC and mGMYC. GMYC analyses use ML and an ultrametric tree to find branch patterns to model the difference between speciation events and neutral coalescence within species (Blair & Bryson, 2017). Traditionally, GMYC has been considered a tool with a tendency to over‐split, and therefore, it is useful to find cryptic species when morphospecies are divided into several units (Ceccarelli et al., 2012). This over‐splitting phenomenon has been previously observed in *Gaidropsarus* (Barros‐García et al., 2022). Collapsing sequences to haplotypes is a common approach for GMYC analyses for less‐demanding computational methods, and there is no evidence of different results when non‐collapsed and collapsed datasets are compared (Talavera et al., 2013). However, a high presence of singletons in the data (a single representative for species) will lower the portion of the tree that provides information to the GMYC mode (Lim et al., 2011). Nevertheless, some studies concluded that singletons are not a problem as long as other better represented taxa can allow for the calibration of the divergence (Reid & Carstens, 2012). This might not be the case, as the dataset presented here could show more recent speciation events among some species (i.e., *G. mauritanicus* sp. nov. and *G. gallaeciae*) than others. Moreover, because recent studies have highlighted the presence of unknown diversity in *Gaidropsarus*, we can consider the phylogenetic inference incomplete due to undersampled taxa (Barros‐García et al., 2018; Barros‐García et al., 2022; Biscoito & Saldanha, 2018). Therefore, the results obtained with GMYC and mGMYC should be interpreted cautiously and compared with separate analyses.

Of particular note is that from all genetically studied samples, *G. mauritanicus* sp. nov. is genetically most closely related to a species from the Huon Marine Park, Tasmania (43°58′ S, 147°32′ E) from 180 to 237‐m depth (Process ID FOAO845‐15) (Figures 5 and 8), which is from biogeographical point the most distant among the studied species. So far, two species could potentially be considered for this haplotype, especially—with regard to depth—*G. novaezealandiae* (300–500 m) and *G. parini* (310–610 m) could be potential species of the undetermined sample. Looking more closely at the coloration of the animal after it was frozen for 8 years (personal communication J. Pogonoski 31.08.2023) (Figure 8a), one can see a golden body coloration with many dark pigments on the dorsal side of the animal and comparatively smaller ones toward the lateral and the ventral sides. This coloration is very similar to the coloration of an individual identified and photographed as *G. novaezealandiae* in Fishes of Australia (*Gaidropsarus novaezealandiae* in Fishes of Australia, accessed September 11, 2023, https://fishesofaustralia.net.au/home/species/2856). However, no reliable identification can be made based on coloration alone. The individual also has 46 vertebrae, suggesting *G. novaezealandiae* instead of *G. parini*. Further, an individual with chocolate brown skin coloration identified as *G. novaezealandiae* is listed in the Natural History Museum collection with the collection number 2019.8.15.4 (see https://data.nhm.ac.uk/object/15c6390c-230e-4b8f-b575-111e7084fd4c/1694563200000; Retrieved: September 13, 2023 11:26:45 a.m. (UTC)). It was collected in a rock pool near the harbor of Tristan da Cunha Island (37°03′51″ S, 12°18′45″ W). However, a chocolate brown coloration from *G. novaezealandiae* was additionally reported by Svetovidov (1986b) from an individual preserved in ethanol. Therefore, a detailed morphometric analysis will clarify its species affiliation in the future.

However, apart from the Tasmanian sample, there are no publicly available genetic data on *Gaidropsarus* species from the Southern Hemisphere. Future studies might clarify a potential influence by southern oceanographic features, such the Antarctic Circumpolar Current, as “highways” for larvae to the Northern Hemisphere. The second genetically closely related species is *G. gallaeciae*, which morphologically resembles *G. mauritanicus* sp. nov. very closely. Its close relation could be explained by the distribution of the poleward undercurrent flow (PUC) (see e.g., Versteegh et al., 2022).

### 5.2 Biogeographical distribution, habitat, and behavior of *G. mauritanicus* sp. nov.

In addition to the holotype sampled in the Tanoûdêrt Canyon, 22 individuals were documented during ROV dive analyses off Mauritania, including the southern flank of the Tanoûdêrt Canyon (station GeoB 14796‐1). The upper slope and outer rim of the canyon shoulder feature the largest and most flourishing coral reef constructions observed during the entire MSM 16/3 expedition. The Tanoûdêrt Canyon System (TanCS) is the northernmost Mauritanian canyon system—situated off Banc d'Arguin, about 70 km southwest of Cape Blanc. The TanCS is included in an exceptionally productive area, belonging to the along‐slope upwelling region (Hernández‐León et al., 2007; Peña‐Izquierdo et al., 2012). These canyons constitute the northern limit of the most productive area from the Mauritanian slope, called the “tongue of the Banc d'Arguin,” where very high values of density and biomass of fishes and cephalopods are located in a continuous band that extends from the shelf to the deep slope (Ramos et al., 2017), thus making this a site the most promising region to encounter *G. mauritanicus* sp. nov. However, just three individuals could be documented during the ROV dive in the Tanoûdêrt Canyon, supporting our presumption that this methodology does not reveal the existing amount due to the high habitat complexity in combination with the sheltering behavior of the species. Therefore, we have not conducted any statistical analysis on the observation data and have chosen to remain on a descriptive level to characterize its distribution, habitat, and behavior.

One of the individuals was observed with whitish colored marks on its skin and was the only individual associated with a branched, live bryozoan of whitish color (*Celleporina* cf. *lucida*), grown on a dead coral framework fragment. The distinctive marks, in addition to the preferred resting behavior of the species in protective places, might indicate territoriality for this species. The protective habitat of *G. mauritanicus* sp. nov., combined with its “guarded” behavior, could be why this species has remained undescribed until now, and why we are unable to add paratypes to the species description.

Deep‐water coral ecosystems are known as biodiversity hotspots in the deep, although only a few fish species are known to be exclusively associated with these ecosystems. Despite this, high numbers of gravid female redfish (*Sebastes norvegicus* [Ascanius, 1772]) on Norwegian *D. pertusum* reefs imply that deep‐water coral ecosystems may provide an essential habitat as spawning sites or nursery areas for juveniles (Costello et al., 2005; Husebø et al., 2002). It is possible that the three‐dimensional structure of a deep‐water coral habitat allows a variety of animals to use these structures to find hiding places from predators or to raise their offspring protected and close to food. As these habitats can usually only be studied at great expense due to the need for specialized research vessels and specific sampling gear (Caiger et al., 2021), little is known about deep‐water coral habitats and their inhabitants compared to shallow‐water coral habitats. There are still huge gaps in our knowledge, especially in the behavior and ecology of such deep‐water coral‐associated species.

Biscoito and Saldanha (2018) report from *G. mauli* an opportunistic feeding behavior, which is supported by the analysis of gut contents collected from several individuals. The guts contained crustacean and fish, which were known to live in close association with hydrothermal vents and also with deep‐water coral reef‐associated fauna. We presume a similar feeding habit for *G. mauritanicus* sp. nov. living in deep‐water coral ecosystems. An opportunistic feeding habit and protection‐seeking behavior might also be supported by its elongated body. Additionally, there is a potential sensitivity gain in having an elongated body, and this may be an important factor in the evolution of elongated bodies (Priede, 2017), including those seen in the genus *Gaidropsarus*. However, the genus is also characterized by an interrupted lateral line. Whether or not these interruptions of the lateral line are useful for perception cannot be answered with the current level of knowledge and requires further investigation.

### 5.3 Distribution and genetic relationship of *Gaidropsarus*

Members of *Gaidropsarus* inhabit a remarkable variety of ecosystems in both the Northern and Southern Hemispheres. They range from shallow‐water areas to deep‐sea hydrothermal vent fields and coral ecosystems, and from the Arctic to subtropical marine ecosystems. In Figure 9, all genetically validated *Gaidropsarus* records with published sampling coordinates are mapped (see Appendix; Table S1). So far, two boreal species (*G. argentatus* and *G. ensis*) have been identified genetically in the western North Atlantic, partly co‐occurring and both with broad bathymetric distributions (Table 3). *G. argentatus* dominates in Arctic waters and *G. ensis* in Labrador waters (see Figure 9b). Genetically, the closest relative of the boreal species is a *Gaidropsarus* specimen sampled from the deep northern Bay of Biscay (from approximately 1229‐m depth), recently identified as *G. mauli* (Bold‐ID: GSRUS016‐16, Collection‐ID: MNHN‐IC 2004‐1517). So far, this record represents the only *COI* sequence of *G. mauli*, but the species was further reported from the Lucky Strike vent site on the Mid‐Atlantic Ridge and the Blackmud Canyon at the slope of the Bay of Biscay, however, to date without any further genetic data (Biscoito & Saldanha, 2018). A comparison of the genetic data indicates that ancestors of *G. mauli* might have migrated to the Northwest Atlantic, resulting in the two boreal species over time. This would imply a palaeoceanographic situation that has enabled an east–west biogeographic spread, opposite to the present situation in the Bay of Biscay (e.g., Mulder et al., 2011; Pingree, 1973; Van Aken, 2000).

Genetically verified records of the eurybathic species *G. macrophthalmus* depict a wide biogeographical distribution in the northeastern Atlantic, mainly occurring in the Mediterranean outflow water. *G. macrophthalmus* is further present in the Mediterranean Sea and in the Black Sea. It is worth noting that its Black Sea records are still listed in BOLD systems as *G. mediterraneus*. In contrast, the genetically verified distribution of *G. mediterraneus* is so far just reported from to the coastal waters off the Azores (Portugal), off Concarneau (France), and off Galicia (Spain), at the latter two sites co‐occurring with *G. vulgaris*. Due to species delimitation analyses, we transferred all records for *G. mediterraneus* from the Mediterranean Sea to the species *G. macrophthalmus* (see Figure 5 and Appendix Table S1–S3). The current identifications of *G. mediterraneus* and *G. macrophthalmus* might have used the first available sequences as a reference, and if these were incorrectly identified, this could have a negative impact on further genetic identifications. Genetic data would therefore be biased and could explain the differences in the representation of the distribution of the two species. The lack of *G. mediterraneus* individuals plotting in the Mediterranean and Black Sea can also be a sampling bias; however, both species appear similar in coloration at first glance based on the literature (Bañón et al., 2022; Cohen et al., 1990). Therefore, it is important to refer to other morphological characteristics when differentiating between *Gaidropsarus* species, for example, the eyes in *G. macrophthalmus* are much larger (“eye large, more than half the snout length” [Cohen et al., 1990; Svetovidov, 1986a]) than those of *G. mediterraneus*. In addition, *G. macrophthalmus* has “enlarged” canine teeth compared to *G. mediterraneus*. To address this question in future research and validate the publicly available sequences, type material of both species should be used for additional sequencing. This would ensure that the sequences indeed correspond to the respective species. The second verified species from the Mediterranean Sea is *G. granti*. Unfortunately, only few records of this species exist, and they all plot in the bathyal Alboran Sea near deep‐water coral occurrences (see also Freiwald et al., 2021).

Our depth extraction from GEBCO (2023) for all records shows that *G. mediterraneus* and *G. vulgaris* have been so far just verified from coastal shallower zones down to 120 m (see Table 3). For *G. vulgaris* the northernmost, genetically verified record is Kattegat.

Furthermore, the coloration of *Gaidropsarus* individuals, which have previously been studied, shows a clear visual separation between dark skin with bright mottled markings for *G. mediterraneus* (including the synonym *G. guttatus*) in contrast to bright skin with or without darker (mainly brownish) mottled markings for the rest. This visual separation is also mirrored in the genetic relationship of the species analysed (see Figure 5). In addition, *G. macrophthalmus* is most closely related to *G. vulgaris* and *G. granti*, and all are characterized by a bright skin with strong or light darker mottled markings. The boreal species all share a strong pinkish skin coloration, and *G. gallaeciae* and *G. mauritanicus* sp. nov. possess pinkish skin. For some *Gaidropsarus* species, juvenile individuals can be colored differently than adults, which holds also for *G. mauritanicus* sp. nov.

### 5.4 Ecology of deep‐water *Gaidropsarus* species

The deep‐water ecology of the genus *Gaidropsarus* presents a complex picture, consisting of reports from various regions. Associations with framework‐forming deep‐water coral ecosystems are reported from several species, including *G*. cf. *vulgaris* in the Celtic Sea (Beuck, 2008; Duineveld et al., 2007; van Oevelen et al., 2018) and *G. gallaeciae, G. vulgaris, G. granti*, and *Gaidropsarus* sp. in the South European Atlantic Shelf (Altuna, 2012; Bañón et al., 2020; Bañón et al., 2022; González‐Irusta et al., 2021; Ríos et al., 2018). Further records are published from the Mediterranean Sea, in particular *G. granti* and *G. macrophthalmus* from the western Mediterranean, *G. granti* from the Adriatic Sea, and *G. macrophthalmus* from the Ionian Sea (Angeletti et al., 2014; Arena & Li Greci, 1973; Bo et al., 2012; Bo et al., 2020; D'Onghia et al., 2010; Massuti et al., 2022; Mastrototaro et al., 2010; Panetta et al., 2012). Additionally, *G. ensis* has been reported from the northwestern Atlantic subarctic (Durán Muñoz et al., 2012), and from the Gulf of Maine/Bay of Fundy, *G. argentatus* and *G. ensis* have been documented (Quattrini et al., 2015).

Beyond this, *Gaidropsarus* individuals have been observed using ROV in deep‐water ecosystems, such as seamounts and deep‐water coral ecosystems near hydrothermal vent fields. However, sampling has often been challenging due to the sheltering behavior of these species. Predictions by Bañón et al. (2022) and Barros‐García et al. (2018), Barros‐García et al. (2022) suggest the existence of several undescribed *Gaidropsarus* species in deep‐water habitats and a decline in shallow‐water species due to synonymy. The first theory is also supported by our ROV observations from deep‐water coral ecosystems (see Figure 10), where a species affiliation based on ROV footage in some cases failed. Furthermore, an identification of juvenile individuals posed a particular challenge, such as for juveniles sampled in association with the interstices of *Madrepora oculata* colonies in the Bari Canyon of the Mediterranean Sea (Le Guilloux, 2011, see also Figure 10g,h). Although only *G. macrophthalmus* is traditionally considered a deep‐sea species in that area (e.g., Cohen et al., 1990), morphological differences from known pelagic juveniles of *G. mediterraneus* and *G. macrophthalmus* (former *G. biscayensis*) raised questions (see Le Guilloux, 2011). Nevertheless, the presence of juveniles in deep‐water coral frameworks highlights the importance of these ecosystems as potential spawning and nursery grounds for fish. Interestingly, Beuck (2008) also noted *Gaidropsarus* from a benthic lander study on the Galway Mound (see also Hebbeln et al., 2004), where an individual was observed remaining for an entire tide cycle in a depression below protective scleractinian framework canopies (see also Figure 10a,b). Linley et al. (2017) also observed a *Gaidropsarus* individual in close association with deep‐water coral ecosystems in the Porcupine Seabight. These observations support the hypothesis that many deep‐water *Gaidropsarus* are closely associated with deep‐water coral ecosystems during their entire ontogeny (see also Figure 10d–f). Furthermore, the presence of individuals with mottled markings dorsally (Figure 10a,b) or laterally (see Figure 10c) suggests a form of camouflage when hiding below or next to coral frameworks, potentially providing protective advantages, especially for species with territorial tendencies.

## 6 CONCLUSION

The existence of *G. mauritanicus* sp. nov. as a new species is morphologically confirmed by the presence of only 44 vertebrae, a conspicuously long pelvic fin (35.7% SL), the large eyes (38.1% HL), medium‐sized pectoral fin (13.8% SL), and the pinkish coloration with several brighter dots along the base of the dorsal fin, as well as genetically confirmed by several independent species delimitation analyses. Furthermore, ROV and lander surveys reveal insights into the ecology and behavior of deep‐water coral‐associated *Gaidropsarus* species, suggesting that these species are territorial and protective. Genetically supported distribution data show clear patterns in the biogeographical and bathymetrical distribution of the different *Gaidropsarus* species. Recent and ancient oceanography seems to be an important factor in terms of larval distribution and phylogenetic relationships, such as the first evidence of *G. mauli* as a basal member of the boreal *Gaidropsarus* species. Further, the genetic and distribution data also indicate possible misidentifications between *G. macrophthalmus* and *G. mediterraneus*. This highlights the urgent need for a comprehensive taxonomic identification key and a complete scientific collection. There still remains a great knowledge gap concerning the *Gaidropsarus* species of the Southern Hemisphere, which should be the focus of future investigations.

## AUTHOR CONTRIBUTIONS

All authors contributed to the study design, analysis, description of the results, writing and proofreading. Alex H. Knorrn described the specimen, collected data and wrote the initial manuscript. Lydia Beuck collected the holotype, carried out the post‐processing of the micro‐CT data, performed the data analysis and generated the distribution maps. David Barros‐García conducted the species delimitation analysis. Lourdes Fernández Peralta conducted the DNA barcoding of the holotype. André Freiwald collected the holotype and supervised the study.

## FUNDING INFORMATION

This study was funded by the GIZ WASP (West African Biodiversity under Pressure) Project (Contract 81248171) to AF and the ERA‐Net Cofund BiodivRestore Project RESTORE SEAS funded by the Deutsche Forschungsgemeinschaft (FR 1134/21‐1). David Barros‐García was supported by national funding from FCT—Foundation for Science and Technology (10.54499/2020.04364.CEECIND/CT0008). Additional strategic funding was provided by FCT UIDB/04423/2020 and UIDP/04423/2020.

## Supporting information


**Data S1.** Supporting information.

**Video S1.** MSM16‐3_5_12–43_Gaidropsarus‐mauritanicus_swimming.

**Video S2.** MSM16‐3_9_14‐13_Gaidropsarus_mauritanicus resting_and_hiding.
